# Heat Stress-Responsive Transcriptome Analysis in the Liver Tissue of Hu Sheep

**DOI:** 10.3390/genes10050395

**Published:** 2019-05-22

**Authors:** Yaokun Li, Lingxuan Kong, Ming Deng, Zhiquan Lian, Yinru Han, Baoli Sun, Yongqing Guo, Guangbin Liu, Dewu Liu

**Affiliations:** 1 College of Animal Science, South China Agricultural University, Guangzhou 510642, China; liyaokun1986@163.com (Y.L.); 18344308934@163.com (L.K.); dengming@scau.edu.cn (M.D.); 18820425216@163.com (Z.L.); hyr_19941128@sina.cn (Y.H.); baolisun@scau.edu.cn (B.S.); yongqing@scau.edu.cn (Y.G.); 2 National Local Joint Engineering Research Center of Livestock and Poutry, South China Agricultural University, Guangzhou 510642, China

**Keywords:** Hu sheep, heat stress, lncRNA, mRNA, transcriptome analysis

## Abstract

Heat stress has a severe effect on animal health and can reduce the productivity and reproductive efficiency; it is therefore necessary to explore the molecular mechanism involved in heat stress response, which is helpful for the cultivation of an animal breed with resistance to heat stress. However, little research about heat stress-responsive molecular analysis has been reported in sheep. Therefore, in this study, RNA sequencing (RNA-Seq) was used to investigate the transcriptome profiling in the liver of Hu sheep with and without heat stress. In total, we detected 520 and 22 differentially expressed mRNAs and lncRNAs, respectively. The differentially expressed mRNAs were mainly associated with metabolic processes, the regulation of biosynthetic processes, and the regulation of glucocorticoid; additionally, they were significantly enriched in the heat stress related pathways, including the carbon metabolism, the PPAR signaling pathway, and vitamin digestion and absorption. The co-located differentially expressed lncRNA Lnc_001782 might positively influence the expression of the corresponding genes APOA4 and APOA5, exerting co-regulative effects on the liver function. Thus, we made the hypothesis that Lnc_001782, APOA4 and APOA5 might function synergistically to regulate the anti-heat stress ability in Hu sheep. This study provides a catalog of Hu sheep liver mRNAs and lncRNAs, and will contribute to a better understanding of the molecular mechanism underlying heat stress responses.

## 1. Introduction

The development of the sheep industry is influenced by a number of factors, including the breeding of different varieties, the selection of the breeding mode, the influence of the climate and environment, and the heat stress, all of which have an important influence on sheep husbandry. The economic impact of heat stress on global livestock production is thought to exceed $1.2 billion [1]. Heat stress has a severe effect on the health of animals and compromises the immune function. It can reduce the productivity and reproductive efficiency of livestock [2], and may eventually lead to multiple organ failure and death [3]. The effects of heat stress are evident in the feed consumption, production efficiency in terms of milk yield or weight gain per unit of feed energy, growth rate, egg production, and reproductive efficiency [4]. In addition, heat stress can cause a decrease in the birth weight of sheep [5,6], and lead to an increase in the rectal temperature and respiratory rate, as well as a decrease in the body weight, average daily gain, and growth rate [7]. Under heat stress, sheep could activate corresponding mechanisms to offset the effects, such as reducing dry matter consumption, and increasing the respiratory frequency and water consumption [8]. Heat resistance is usually measured in view of the respiratory rate and rectal temperature [9]. Based on the air temperature, relative humidity, respiration rate and temperature-humidity index, heat-tolerant models of 11 different breeds of sheep in Brazil have been examined [10]. At present, studies on sheep heat stress mainly focus on physiology, behavior and production. With the rapid development of transcriptome and proteomics studies, the molecular mechanism of heat stress regulation on sheep has been studied, but a deeper and more systematic study on the molecular mechanism still needs to be carried out. The transcriptome studies on heat stress mainly focus on dairy cows, dairy goats, pigs, chickens, and rainbow trout. A large number of genes and regulatory pathways related to heat stress have been identified in these species through transcriptomic analyses. In dairy cows, the increased expression of heat shock proteins (HSPs), which prevent protein aggregation and degrade misfolded proteins, was identified in heat-stressed animals, and several genes involved in the fatty acid metabolism were also differentially expressed; under heat stress conditions, ACOT7 (involved in long-chain fatty acid hydroxylation), CPT1C (involved in fatty acid β oxidation), and ATF4 (regulates fatty acid synthesis) were upregulated, while RAC1, CDC42 and PRKAB2 (regulate key enzymes involved in the de novo biosynthesis of fatty acids) were downregulated [11]. The heat stress related study on dairy goats showed that the differentially expressed genes (DEGs) were mainly enriched in the Gene Ontology (GO) terms related to biological processes and, to a lower extent, to molecular functions and cellular components. Moreover, the Ingenuity Pathway Analysis (IPA) detected important pathways related to cell proliferation and death, free radical scavenging, inflammatory response, lipid metabolism, and glycolysis/gluconeogenesis; the transcriptional regulators most strongly affected by heat stress were: SATB1 (global chromatin organizer) and PPARD (might be related to insulin resistance) [12]. Additionally, 78 differentially expressed genes were identified in the porcine longissimus dorsi muscle under constant heat stress, and these were mainly involved in muscle development, glycolysis, lipid metabolism and stress response; in the group under heat stress, the DEGs involved in the glycolysis and lipid metabolism pathways, including TPI1, GPI, PFKM, PGAM2, PKM2, LDHA, PGK1, ENO3, FASN, and SCD, were down-regulated; the DEGs predicted to play roles in the heat stress response (EIF4A2, RPL15, CRYAB, HSP90AB1, and HSPB1) and oxidative metabolism (COX1, 2, and 3; ND1, 2, and 5; and PDK4) were up-regulated [13]. A transcriptomic analysis in chickens revealed that the transcripts of heat-shock proteins, including Hsp40 and Hsp90, were significantly altered in response to thermal stress [14]. The transcriptome analysis in the rainbow trout identified a large number of DEGs associated with heat stress, including HSP47, HSP90, and HSP70; and many novel genes such as CYPLA3, CCT3, and PDIA3 were implicated in the thermal acclimation process [15]. However, rare research on the combination of lncRNA and heat stress has been reported, particularly in sheep. The liver is the most crucial metabolic organ associated with stress response; however, little is known about the mechanism underlying heat stress response in sheep. Therefore, in this study, we performed a transcriptomic analysis of Hu sheep liver following exposure to heat stress to identify related mRNAs, lncRNAs and pathways. Our results will facilitate the identification of critical genes and pathways that are involved in the regulation of the heat stress response. The genes identified in this study could serve as candidates for the development of breeding programs to produce sheep varieties with a superior heat tolerance.

## 2. Methods

### 2.1. Ethical Statement

The study was approved by the Ethics Committees of the Laboratory Animal Center of South China Agricultural University (permit number: SYXK-2014-0136). All experiments were performed in accordance with the South China Agricultural University guidelines.

### 2.2. Animals and Sample Collection

Female Hu sheep were born, raised and maintained at the same farm. All animals included in this study received the same diet until they were slaughtered. All experimental animals were fed the same diet throughout the year. The feedstuff mainly included silage, hay and concentrate, which were mixed by total mixed rations (TMR) machine and fed twice a day. The experimental animals were divided into two groups: with heat stress (75 ≤ temperature humidity index (THI) ≤ 85) and without heat stress (THI < 75). The THIs were measured with a wet-bulb and dry-bulb recording thermometer; the data were collected three times a day. The formula of the THI calculation was: THI = 0.72 (Td + Tw) + 40.6, of which Td and Tw were the dry ball index and wet ball index, respectively. In addition, the frequency of breathing and the rectal temperature were the most significant indicators of whether and to what extent the animal itself experienced heat stress, and were also recorded. 20 days before the sampling, we collected the THI, the animals’ breathing rate and the rectal temperature. In summer, when 75 ≤ THI ≤ 85, the average breathing rate was 101.54 times/min, and the mean rectal temperature was 39.38 ℃; we randomly chose three sheep as the heat stress group. In autumn, when THI < 75, the average breathing rate and rectal temperature were 48.55 times/min and 38.56 ℃, respectively; another three sheep were randomly selected and assigned to the group without heat stress. All the six experimental animals were healthy and almost the same age (1.4~1.6 years old); when they were slaughtered, the length of the wool was similar. Before slaughter, we performed a jugular vein blood collection for each experimental animal, separated the serum, and measured the blood biochemical indexes, including: calcium (Ca), sodium (Na), potassium (K), total protein (TP), urea nitrogen (BUN), creatine kinase (CK), triidothyronine (T3), tetraiodothyronine (T4), glucose (GLU), corticosterone (Cor), superoxide dismutase (SOD), heat shock protein 70 (HSP70) and lactate dehydrogenase (LDH); after slaughter, the liver tissue was collected. The liver samples were collected at the same site from each experimental animal and immediately frozen in liquid nitrogen for a total RNA extraction after harvesting.

### 2.3. cDNA Library Preparation

The total RNA was isolated using a TRIzol reagent (Invitrogen, Carlsbad, CA, USA) and treated with DNase I (Qiagen, Beijing, China). The purified RNA was assessed by 1.5% agarose gel electrophoresis to confirm the absence of any genomic DNA contamination. The RNA integrity was assessed using the RNA Nano6000 Assay Kit and the Bioanalyzer 2100 system (Agilent Technologies, Santa Clara, CA, USA).

The total RNA (3 μg) was used as input material for each sample preparation. First, the ribosomal RNA (rRNA) was removed using the Epicentre Ribo-zero™ rRNA Removal Kit (Epicentre, Madison, WI, USA), and the rRNA-free residue was cleaned up by ethanol precipitation. Subsequently, sequencing libraries were generated using the rRNA-depleted RNA with the NEBNext® Ultra™ Directional RNA Library Prep Kit for Illumina® (NEB, Beverly, MA, USA), according to the manufacturer’s instructions. Briefly, fragmentation was carried out in NEBNext First Strand Synthesis Reaction Buffer (5×). First strand cDNA was synthesized using random hexamer primers and M-MuLV reverse transcriptase (RNaseH). The second strand cDNA synthesis was then performed using DNA polymerase I and RNaseH. In the reaction buffer, dNTPs with dTTP were replaced by dUTP. The remaining overhangs were converted into blunt ends via exonuclease/polymerase activities. After the adenylation of the 3′-ends of the DNA fragments, NEBNext Adaptors containing a hairpin loop structure were ligated to prepare for the hybridization. To preferentially select cDNA fragments of 150–200 bp in length, the library fragments were purified using the AMPure XP system (Beckman Coulter, Miami, FL, USA). Subsequently, 3 μL USER enzyme (NEB, Beverly, MA, USA) was incubated with size-selected, adaptor-ligated cDNA at 37 °C for 15 min, followed by 5 min at 95 °C. A PCR amplification was then performed with Phusion High-Fidelity DNA polymerase, Universal PCR primers and Index (X) Primer. Finally, the products were purified using the AMPure XP system (Beckman Coulter, Miami, FL, USA), and the library quality was assessed using the Agilent Bioanalyzer 2100 system (Agilent Technologies, Santa Clara, CA, USA).

### 2.4. Sequencing and Transcriptome Assembly

The constructed libraries were sequenced on an Illumina HiSeq 4000 platform, and 150-bp paired-end reads were generated. After removing the sequence containing adapter and the reads containing ploy-N and low-quality reads through in-house Perl scripts, clean data were obtained. All the downstream analyses were based on the clean data with high quality. To obtain the high-quality reads, we performed the following filtering process: we removed the reads containing more than 10% unknown nucleotides and the reads containing more than 50% low quality nucleotides with Phred with a quality under 20. Mapping to the reference genome was the next step. Reads that passed the quality control were then mapped to the *Ovis aries* reference genome (Oar_v3.1). The index of the reference genome was built using bowtie2 v2.2.8, and paired-end clean reads were aligned to the reference genome using HISAT2 (v2.0.4) [16]. HISAT2 was run with “--rna-strandness RF”, and other parameters were set as default. Next was the transcriptome assembly. The mapped reads of each sample were assembled by StringTie (v1.3.1) [17]. Our sequencing data could be found in the sequence read archive (SRA) of NCBI. The submission ID was SUB4512950, and the bioproject ID was PRJNA490799.

### 2.5. Prediction of the Differentially Expressed mRNAs and Long Non-Coding RNAs

Before the screening, Cuffmerge was used to create the set of transcripts. Then, the lncRNA screening was carried out through the following steps: Step1: select the number of transcripts with ≥2 exons; Step2: out of the results from step1, select the transcripts with a length >200 bp; Step3: annotate the above transcripts using the Cuffcompare software; Step4: calculate the expression level of each transcript by Cuffquant, and select the transcripts with FPKM ≥ 0.1; Step5: coding the potential screening: the coding potential of the transcript was predicted by three softwares: CNCI (Coding-Non-Coding-Index) (v2) [18], CPC (Coding Potential Calculator) (0.9-r2) [19], and PFAM (Pfam Scan) (v1.3) [20,21]; the intersection of the transcripts without a coding potential screened through the above three softwares with the default parameter was predicted as the lncRNA dataset.

The Ballgown was utilized to perform the straightforward linear-model-based differential expression analyses within a default statistical modeling framework [22,23]. The transcripts with *p*-adjust < 0.05 were assigned as being differentially expressed.

### 2.6. Target Gene Prediction of the lncRNAs

For each lncRNA locus, the 100-kb upstream and downstream regions were chosen to screen the co-located genes through the UCSC Genome Browser [24].

### 2.7. GO and KEGG Enrichment Analysis

A Gene Ontology (GO) enrichment analysis of the DEGs or lncRNA target genes was implemented using the GOseq R package, in which the gene length bias was corrected [25]. The GO terms with a corrected *p* < 0.05 were considered significantly enriched for differential expressed genes.

KEGG (Kyoto Encyclopedia of Genes and Genomes) was a database resource for understanding the high-level functions and utilities of biological systems [26], such as the cell, organism and ecosystem, from molecular-level information, especially large-scale molecular datasets generated by genome sequencing and other high-throughput experimental technologies (http://www.genome.jp/kegg/). We used KOBAS software to analyze the enrichment of the DEGs or lncRNA co-located genes in the KEGG pathways [27].

### 2.8. Quantitative Real-Time Polymerase Chain Reaction Validation

For the qRT-PCR analysis, 1 µg of total RNA was reverse transcribed using the RT reagent Kits with gDNA Eraser (Takara, Dalian, China), according to the manufacturer’s protocol. The qRT-PCR was performed on a StepOnePlus Real-Time PCR System (Life Technologies, Gaithersburg, MD, USA), according to standard methods using Fast Start Universal SYBR Green Master (ROX) (Roche, Mannheim, Germany).

## 3. Results

### 3.1. Blood Biochemical Parameters

The measurement results of the blood biochemical are shown in Table 1. Compared with the WHSHS group, the concentrations of LDH and T4 in the HSHS group increased significantly, and the Na^+^ concentration decreased significantly (*p* < 0.01). Additionally, the biochemicals TP, SOD and cortisol increased significantly, and the concentration of HSP70 decreased significantly in the HSHS group (*p* < 0.05). The other biochemical indexes showed no significant difference between the two experimental groups (*p* > 0.05).

### 3.2. Read Mapping

Generally, the proportion of the nucleotides with Q30 in the total nucleotides should be larger than 85%; in our study, the proportion of each sample exceeded 91%. In total, 86,231,172, 80,485,006, 84,145,628, 85,369,917, 72,563,302, and 83,970,873 mapped reads of the HSHS03, HSHS06, HSHS36, WHSHS08, WHSHS48, and WHSHS67 libraries, respectively, were obtained from the clean data, and more than 85% were mapped to the *O. aries* reference genome (Table 2).

### 3.3. Enrichment Analysis of the Differentially Expressed mRNAs

**Figure 1:**
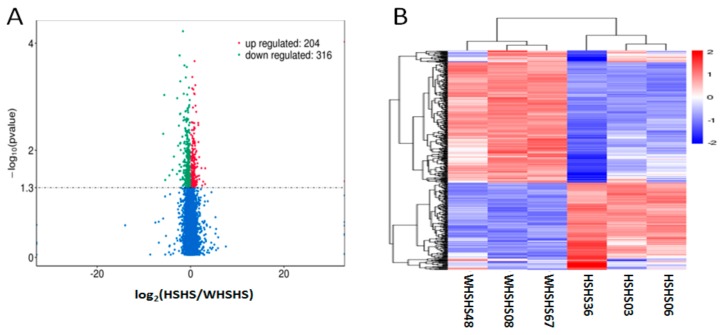
Analyses of differentially expressed mRNAs in the RNA-seq libraries. (**A**) Volcano plot showing the overall distribution of the differential transcript or gene, with the threshold set to *q* < 0.05. Red: the genes significantly upregulated in the heat stress group (*p* < 0.05); Green: the genes significantly downregulated in the heat stress group (*p* < 0.05). (**B**) Hierarchical clustering analysis of mRNA expression profiles with 520 differentially expressed mRNAs.

The transcripts with *p* < 0.05 were classified as differentially expressed. In total, we identified 520 mRNA transcripts that were differentially expressed. Compared with the WHSHS library, 204 mRNAs were upregulated in the HSHS library and 316 mRNAs were downregulated (Figure 1A and Additional file 1). To explore the similarities and to compare the relationships between the different libraries, the expression pattern of the differentially expressed mRNAs was evaluated by a systematic cluster analysis (Figure 1B). To further clarify the biological function, we carried out a GO and KEGG enrichment analysis of the 520 differentially expressed mRNAs. In total, we found 50 GO terms with *p* < 0.05 (Additional file 2), most of which were associated with a biological process, including metabolic processes, the regulation of biosynthetic processes, and the regulation of glucocorticoid receptor signaling pathways (Table 3). Additionally, we detected 17 KEGG pathways significantly enriched by the differentially expressed mRNAs (*p* < 0.05) (Table 4 and Additional file 3), several of which were related to heat stress, including the carbon metabolism, metabolic pathways, biosynthesis of amino acids, the PPAR signaling pathway, and vitamin digestion and absorption. Of the top 50 DEGs, NR1H3 was involved in the PPAR signaling pathway, and it also showed an association with the biological process of the cellular metabolic process, nitrogen compound metabolic process, and biosynthetic process; APOA4 was discovered in the pathway of vitamin digestion and absorption, and this gene was related to the cellular metabolic process, fatty acid biosynthesis process, fatty acid metabolic process, and fatty acid biosynthetic process; both ALDOB and ETNK1 were associated with metabolic pathways, and they were simultaneously detected in the GO term of the cellular metabolic process.

### 3.4. Enrichment Analysis of the Differentially Expressed lncRNAs

In total, we identified 20 lncRNA transcripts that were differentially expressed (Additional file 4); the sequence could be found in the Additional file 5. Compared with the WHSHS library, 11 lncRNAs were upregulated in the HSHS library, and nine lncRNAs were downregulated (Table 5).

To investigate the possible function of the differentially expressed lncRNAs, we predicted the potential targets of lncRNAs in a cis-regulatory relationship. We searched for protein-coding genes 100-kb upstream and downstream of the differentially expressed lncRNAs. In total, we identified 57 protein-coding neighbors corresponding to the differentially expressed lncRNAs (Additional file 6). GO analysis of the 57 genes was performed to explore their functions. We found 25 GO terms that were significantly enriched (*p* < 0.05) (Additional file 7), and all of these terms were assigned to the biological process. The top 10 enriched terms were mainly associated with the oxygen species metabolic process, regulation of growth, and triglyceride catabolic process (Table 6). Additionally, the co-located genes (INSR, ENSOARG00000011276 and GTF2IRD1) of Lnc_003603 and Lnc_004811 were found to be significantly enriched in the cGMP-PKG signaling pathway (*p* < 0.05), however, none of the three genes were differentially expressed between the two experimental groups.

### 3.5. Combined Analysis of the Differentially Expressed lncRNAs and Differentially Expressed mRNAs

Interestingly, we found three differentially expressed genes in the vicinity of the differentially expressed lncRNA Lnc_001782, including ZPR1, APOA4 and APOA5; furthermore, Lnc_001782 was located between APOA4 and APOA5. ZPR1 was annotated with growth related GO terms, including “regulation of multicellular organismal process” (GO: 0051239), “regulation of growth” (GO: 0040008), “cellular metabolic process” (GO: 0044237), and “biosynthetic process” (GO: 0009058). APOA4 and APOA5 were enriched in “oxoacid metabolic process” (GO: 0043436), “organic acid metabolic process” (GO: 0006082), “regulation of fatty acid biosynthetic process” (GO: 0042304), and “cellular metabolic process” (GO: 0044237). The pathway analysis showed that APOA4 and APOA5 were respectively enriched in vitamin digestion and absorption, and in the PPAR signaling pathway (*p* < 0.05).

### 3.6. Validation of Differentially Expressed lncRNAs and mRNAs

**Figure 2:**
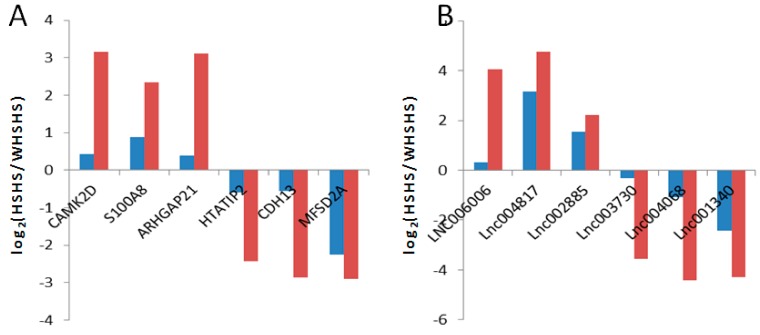
Validation of the differentially expressed mRNAs and lncRNAs by qPCR. (**A**) The qRT-PCR results of the differentially expressed mRNAs were compared with the RNA-Seq. Red: RNA-Seq; Blue: qRT-PCR. (**B**) The qRT-PCR results of the differentially expressed lncRNAs were compared with the RNA-Seq. Red: RNA-Seq; Blue: qRT-PCR.

To further evaluate the RNA-Seq data, six differentially expressed lncRNAs and six differentially expressed mRNAs were randomly selected for a qRT-PCR analysis. The results showed that the gene expression patterns were concordant with the RNA-Seq data, although the absolute fold changes in the expression levels differed between the two sets of data (Figure 2).

## 4. Discussion

The accumulating evidence indicated the important roles of mRNAs in heat stress in chicken, duck, and mice; for instance, three zinc ion-binding genes (GLIS2, ZBTB7A, and TRAF4), MAP3K6, and p38MAPK were highly expressed in the heat stress group compared with the control group [28,29,30]. An improved understanding of these changes will be useful in the development of breeding programs aimed at generating highly heat-resistant animals to reduce the risk of heat stress. Most of the previous bioinformatics analyses revealed a differential mRNA expression in the liver. However, no studies on the function of lncRNAs in regulating the heat stress response in Hu sheep have been reported. Therefore, in this study, we performed transcriptome sequencing of the liver of Hu sheep with and without heat stress and analyzed the differentially expressed mRNAs and lncRNAs to clarify their roles in heat stress, which would be helpful for providing a valuable catalog of functional mRNAs and lncRNAs associated with heat stress in Hu sheep.

Heat stress can affect the metabolism of animals. It was suggested that heat stress could cause changes in the carbohydrate metabolism in cows [31,32]. Compared with the non-heat stressed group, heat stress resulted in a 200–400 g/d decrease in the lactose content in milk produced by Holstein cows [33], which may be due to an abnormal liver function caused by heat stress. In cows, heat stress resulted in increased plasma levels of urea nitrogen, which was the product of protein breakdown and metabolism. A series of studies have confirmed that heat stress could lead to the reduction in the plasma levels of non-esterified fatty acids (NEFA) in animals [34,35,36]. The ability to better absorb and store triglycerides in the intestines and liver could be improved under the condition of heat stress [36]. Ronchi et al. hypothesized that the decrease in plasma NEFA concentrations may be due to the decrease in the fat catabolism caused by heat stress and the improvement of NEFA utilization [37]. In further studies on the inhibition of fat mobilization under heat stress, it was shown that heat stress leads to increased insulin concentrations, which promotes the synthesis and storage of fatty acids and reduces fat decomposition [34]. At the same time, the heat produced by NEFA for β-oxidation (lipid metabolism) was higher than that associated with carbohydrate metabolism, and dairy cows metabolized carbohydrates preferentially to supply energy and reduce their own metabolic heat production. In this study, the VNN1 gene was at the top of the differentially expressed genes; and it was expressed less in animals under heat stress. Many studies showed that VNN1 played a very important role in the lipid metabolism. It was found that the expression of the mouse VNN1 gene could be affected by the fasting and lipid-lowering drug, and could also be regulated by PPARα. PPARα was an important member of the peroxisome proliferator-activated receptor family and was an important regulator of the fat metabolism; it can promote fatty acid catabolism and reduce fatty acid synthesis and storage by regulating the expression of fatty acid-related genes. Therefore, it was suggested that VNN1 might be involved in the body’s lipid metabolism [38,39,40]. VNN1 also played a key role in the glycogenesis pathway, regulating the process of the glucose metabolism. Its altered expression might influence the concentration of LDH in the blood; LDH is found in almost all tissues and is an important enzyme in the body’s metabolic process; it could lead to an enhanced glycolytic process in animals; studies have shown that, with prolonged heat stress, the serum LDH activity increases significantly [41], which was in accordance with our results. The overexpression of VNN1 in mouse hepatocytes could activate glycogenesis and increase the expression of the PEPCK and G6Pase gene and the hepatic sugar output, which was significant for stimulating physiological changes in the body [33], resulting in an elevated concentration of cortisol in heat-stressed Hu sheep blood, for a better resistance to high temperature. The inhibition of the VNN1 expression could improve the tolerance of fasted mice to glucose, reduce the output of liver sugar, and inhibit the expression of gluconeogenesis related genes, which might decrease the heat produced by the body. It was also confirmed that the transcription of VNN1, promoted by the cooperation of PGC-1α and HNF4α, could activate gluconeogenesis, which might be mediated by the Akt signaling pathway [42]. In recent years, studies have shown that the regulation of the VNN1 expression was closely related to liver-related transcription factors (such as PPARs, HNF1, HNF4, C/EBP, etc.) and microRNAs involved in the fat metabolism (such as miR-122, miR-33, miR-370, etc.), indicating that VNN1 had a great potential research value in the process of the liver glycolipid metabolism [42,43,44]. In our study, the expression of the VNN1 gene decreased in the heat stress group, and the biological analysis results revealed that VNN1 was engaged in the GO terms, including “organic acid metabolic process”, “negative regulation of intrinsic apoptotic signaling pathway in response to oxidative stress”, and “T cell differentiation in thymus”. It was suggested that VNN1 might regulate the anti-heat stress process of Hu sheep via the PPAR signaling pathway; its reduced expression would enhance animals’ tolerance to heat stress. Among the top differentially expressed genes, NR1H3 showed a decreased expression level in the animals in summer. It was predicted to be associated with the cellular metabolic process, nitrogen compound metabolic process, and biosynthetic process; the pathway analysis revealed that NR1H3 was significantly enriched in the PPAR signaling pathway. NR1H3 was mainly expressed in the liver, adipose tissue and macrophage, and its main function was to regulate the lipoprotein metabolism and fat synthesis [45,46]. In addition, it could affect the development of pathogenic conditions, including hepatic steatosis, and increase the hepatic triglyceride content; the inhibition of NR1H3 contributes to the recovery from hepatocyte steatosis [47]. Studies have clarified that NR1H3 is the important regulator of hepatic lipogenesis [48]. Thus, we hypothesized that both VNN1 and NR1H3 were involved in the heat stress response, and played a key role in improving animals’ resistance to heat stress; however, the underlying mechanism still required further investigation.

Non-coding genes were distinguished by their potential coding capability. In this study, we used a highly stringent filtering pipeline to minimize the selection of false-positive lncRNAs, with the aim of removing transcripts showing evidence of a protein-coding potential. Using this approach, we identified a total of 6273 lncRNAs. LncRNAs are a group of endogenous RNAs that function as regulators of the gene expression, and are involved in developmental and physiological processes. In total, we detected 20 putative lncRNAs that were differentially expressed in pairwise comparisons between the HSHS and WHSHS libraries, and which might have specific biological roles in heat stress in Hu sheep. These lncRNAs and their functions were all predicted by a bioinformatics method, and further molecular experiments should be carried out for validation using in vitro systems or cell lines, as this would help enrich the functional annotation of the identified lncRNAs; the limitation is that the functional experiments are not included, but we are designing experiments to validate the regulatory function of the discovered lncRNAs. In this study, we identified, in total, 57 protein-coding neighbors 100-kb upstream and downstream of the differentially expressed lncRNAs. A GO analysis showed that these protein-coding genes were associated with the reactive oxygen species metabolic process, growth regulation, triglyceride metabolic process and fatty acid metabolic process; and some of the co-located genes (INSR, ENSOARG00000011276 and GTF2IRD1) of Lnc_003603 and Lnc_004811 were significantly enriched in the cGMP-PKG signaling pathway; this pathway could contribute to the regulation of insulin secretion, influencing the glucose metabolism [49]. Therefore, in addition to mRNAs, the differentially expressed lncRNAs reported here could be considered as important novel regulatory factors involved in the heat stress response in Hu sheep. Among the 20 screened lncRNAs, Lnc_001782 was found to be located between the differentially expressed genes APOA4 and APOA5. APOA4 displayed a significantly lower expression level in the HSHS group. It was predicted to be significantly enriched in the vitamin digestion and absorption pathway, which could potentially enhance the body’s immunity, improving the tolerance to heat stress. This gene can also affect the chylomicron formation and dietary fat absorption [50]. Furthermore, the APOA4 synthesis in the liver and small intestine can be regulated by lipase activity, which could activate lecithin-cholesteryl thiol transferase and affect the lipid metabolism by affecting the cholesterol metabolism. The APOA5 gene was a recently discovered member of the apolipoprotein APOA1-APOC3-APOA4-APOA5 gene cluster, which played an important role in the plasma triglyceride metabolism and lipid-related metabolism [51]. In this study, APOA5 was significantly less expressed in the HSHS group. Previous studies have shown that this gene was associated with high-density lipoprotein cholesterol and plasma triglyceride levels [52]. Lnc_001782, APOA4 and APOA5 were all downregulated in the heat stress group; due to the potential cis-regulatory effect, Lnc_001782 might positively influence the expression of APOA4 and APOA5, exerting co-regulative effects on the liver function corresponding to heat stress.

The heat-stress response is a very complex process, which might be synergistically regulated by our discovered DEGs and differentially expressed lncRNAs, resulting in the altered concentration of blood biochemicals. In this study, we found that Na^+^ was significantly decreased in the blood of animals under heat stress. Na^+^ is an important electrolyte for maintaining the osmotic pressure, acid-base balance, neuromuscular stress and extracellular fluid volume. Previous studies also showed that the Na^+^ concentration would be significantly reduced under heat stress [53]. Compared with the WHSHS group, the HSP70 content was significantly decreased in animals under heat stress; however, many studies suggest that the HSP70 content would be increased due to the stress of a high ambient temperature [54,55]. Perhaps this was related to the reason that our experiment was performed in South China, where most of the time there is a high temperature across the year; and the sheep raised under a high temperature for a long time gradually adapt to this environment; the sudden transition from a high temperature to a low temperature might cause stress to the animals, and the circadian rhythm might also be changed, resulting in an elevated concentration of HSP70 to strengthen the body’s resistance to environmental changes. T4 plays an important role in the animal’s adaptation to environmental changes; it is associated with the basal metabolic rate, glucose utilization, lipid metabolism, and neural functions [56,57]. Normally, the T4 level will decrease under chronic heat stress, trying to reduce the basal metabolism and body temperature [58,59]. However, due to the above reason, the internal environment of the animals sampled in autumn may be disturbed and cause an imbalance of blood biochemicals, resulting in the significant lower level of T4 in the WHSHS group. In this study, we explored the alteration of blood parameters and the liver genetic difference in Hu sheep caused by heat stress. The kidney also plays an important role in animals’ resistance to thermal stress; however, in the current study, we did not study the functional and genetic changes of kidneys, which is a limitation of our research. Additionally, further studies should be carried out to detect the regulative effects of kidneys in the resistance to heat stress.

Accordingly, there might be a possible mechanism by which the lncRNAs could affect the liver function by mediating the regulation of the corresponding target mRNAs. Understanding the mechanism of our screened lncRNAs and mRNAs that might work on the liver function could be useful for animal breeding that involves high heat-resistance characteristics, and this would be significant for relieving heat stress and improving animals’ welfare, while also benefitting animal husbandry.

## 5. Conclusions

In conclusion, the present study provided a systematic description of the changes in mRNAs and lncRNAs in Hu sheep under conditions of heat stress. Furthermore, the data obtained represented a resource for further investigations of the function of some of these lncRNAs, as it was helpful for providing basic information that is required to elucidate the mechanisms associated with the regulation of heat stress in Hu sheep at the molecular level.

## Figures and Tables

**Table 1:** Comparison of the blood biochemical indexes between HSHS and WHSHS.

Biochemical Indexes	HSHS	WHSHS	*p*-Value
CK (U/L)	126.13 ± 38.87	146.27 ± 31.80	0.132
TP (g/L)	71.30 ± 4.33	67.59 ± 3.40	0.014
GLU (mmol/L)	2.23 ± 0.58	2.32 ± 0.33	0.591
BUN (mmol/L)	7.82 ± 1.01	7.30 ± 0.70	0.115
LDH (U/L)	666.27 ± 185.10	441.00 ± 101.74	0.000
Ca (mmol/L)	2.68 ± 0.13	2.67 ± 0.05	0.883
K^+^ (mmol/L)	4.29 ± 0.17	4.21 ± 0.14	0.213
Na^+^ (mmol/L)	141.53 ± 2.38	145.13 ± 1.95	0.000
SOD (U/mL)	102.18 ± 4.68	98.48 ± 4.82	0.042
T3 (ng/mL)	13.10 ± 0.37	13.03 ± 0.39	0.585
T4 (ng/mL)	553.17 ± 9.35	503.56 ± 10.54	0.000
Cor (ng/mL)	16.88 ± 9.97	10.16 ± 6.24	0.035
HSP70 (ng/mL)	15.27 ± 0.81	15.91 ± 0.82	0.038

Note: HSHS: heat stressed Hu sheep; WHSHS: Hu sheep without heat-stress; CK: creatine kinase; TP: total protein; GLU: glucose; BUN: urea nitrogen; LDH: lactate dehydrogenase; Ca: calcium; K: potassium; Na: sodium; SOD: superoxide dismutase; T3: triiodothyronine; T4: tetraiodothyronine; Cor: corticosterone; HSP70: heat shock protein 70.

**Table 2:** Summary of clean reads mapped to the *Ovis aries* reference genome.

Sample	HSHS03	HSHS06	HSHS36	WHSHS08	WHSHS48	WHSHS67
Raw Reads	101,911,032	95,707,656	103,944,564	99,986,482	9,219,4420	104,239,158
Clean Reads	95,803,190	91,642,522	95,317,472	94,234,468	84,488,554	95,701,894
Q30 (%)	92.48	91.71	92.05	92.31	91.67	91.70
GC Content (%)	47.58	46.81	47.15	48.94	49.38	48.20
Total Mapped	86,231,172 (90.01%)	80,485,006 (87.82%)	84,145,628 (88.28%)	85,369,917 (90.59%)	72,563,302 (85.89%)	83,970,873 (87.74%)

Note: HSHS: heat stressed Hu sheep; WHSHS: Hu sheep without heat-stress.

**Table 3:** Biological process enriched by the differentially expressed mRNAs.

Gene Ontology (GO) Term (Biological Process)	Observed *	*p*-Value
Oxoacid metabolic process	45	1.40 × 10^−5^
Organic acid metabolic process	45	1.40 × 10^−5^
Carboxylic acid metabolic process	42	1.40 × 10^−5^
Regulation of fatty acid biosynthetic process	8	4.72 × 10^−4^
Positive regulation of protein deacetylation	6	1.88 × 10^−3^
Alpha-amino acid metabolic process	17	2.60 × 10^−3^
Cellular amino acid metabolic process	23	4.37 × 10^−3^
Small molecule metabolic process	74	4.65 × 10^−3^
Protein deacetylation	10	7.33 × 10^−3^
Regulation of protein deacetylation	7	7.33 × 10^−3^
Protein deacylation	10	8.09 × 10^−3^
Negative regulation of glucocorticoid receptor signaling pathway	4	9.81 × 10^−3^
Cellular metabolic process	251	1.29 × 10^−2^
Small molecule biosynthetic process	22	1.51 × 10^−2^
Regulation of glucocorticoid receptor signaling pathway	4	1.86 × 10^−2^
Organic acid biosynthetic process	17	1.90 × 10^−2^
Carboxylic acid biosynthetic process	17	1.90 × 10^−2^
Regulation of fatty acid metabolic process	8	2.03 × 10^−2^
Single-organism biosynthetic process	22	2.03 × 10^−2^
Organonitrogen compound metabolic process	60	2.56 × 10^−2^
Monocarboxylic acid biosynthetic process	12	3.34 × 10^−2^
Positive regulation of fatty acid biosynthetic process	4	3.34 × 10^−2^
Nitrogen compound metabolic process	163	3.83 × 10^−2^
Fatty acid biosynthetic process	10	3.87 × 10^−2^
Heterochromatin organization	4	3.87 × 10^−2^
Regulation of lipid biosynthetic process	10	4.11 × 10^−2^
Negative regulation of intracellular steroid hormone receptor signaling pathway	6	4.35 × 10^−2^
Histone modification	22	4.35 × 10^−2^
Biosynthetic process	145	4.35 × 10^−2^
Positive regulation of cellular biosynthetic process	56	4.96 × 10^−2^

Note: * Number of differentially expressed genes.

**Table 4:** KEGG pathway enrichment analysis of differentially expressed mRNAs.

KEGG Pathway	Number of Genes	*p*-Value
One carbon pool by folate	5	6.99 × 10^−4^
Carbon metabolism	11	1.17 × 10^−3^
Biosynthesis of amino acids	8	3.92 × 10^−3^
Spliceosome	11	1.15 × 10^−2^
Protein processing in endoplasmic reticulum	12	1.34 × 10^−2^
Glyoxylate and dicarboxylate metabolism	4	1.43 × 10^−2^
Type I diabetes mellitus	6	1.45 × 10^−2^
Metabolic pathways	54	1.69 × 10^−2^
Phagosome	11	2.33 × 10^−2^
Circadian rhythm	4	2.37 × 10^−2^
Graft-versus-host disease	5	2.45 × 10^−2^
Citrate cycle (TCA cycle)	4	2.60 × 10^−2^
PPAR signaling pathway	6	2.91 × 10^−2^
Allograft rejection	5	3.43 × 10^−2^
Vitamin digestion and absorption	3	4.00 × 10^−2^
Cysteine and methionine metabolism	4	4.52 × 10^−2^
Herpes simplex infection	12	4.53 × 10^−2^

**Table 5:** The 20 differentially expressed lncRNAs in the liver of the Hu sheep.

Transcript ID	Gene ID	FPKM (HSHS/WHSHS)	log_2_ (HSHS/WHSHS)	*p*-Value
LNC_005888	XLOC_171029	67.33/30.65	1.135395178	1.80 × 10^−3^
LNC_004811	XLOC_139887	1.43/2.70	−0.914641699	8.78 × 10^−3^
LNC_003332	XLOC_095440	7.80/12.88	−0.723050426	1.12 × 10^−2^
LNC_001340	XLOC_039194	0.21/4.06	−4.299912802	1.14 × 10^−2^
LNC_001712	XLOC_048154	2.81/0.94	1.580309792	1.43 × 10^−2^
LNC_003730	XLOC_106375	0.24/2.81	−3.559291973	1.63 × 10^−2^
LNC_000765	XLOC_023257	0.85/2.74	−1.690587291	1.70 × 10^−2^
LNC_000193	XLOC_005239	10.93/0.00	–	1.73 × 10^−2^
LNC_003439	XLOC_098618	43.58/11.56	1.915191489	2.23 × 10^−2^
LNC_001699	XLOC_047869	6.44/2.94	1.130446672	3.06 × 10^−2^
LNC_006006	XLOC_173090	1.12/11.56	4.062706206	3.27 × 10^−2^
LNC_004068	XLOC_115897	0.18/2.94	−4.411271344	3.30 × 10^−2^
LNC_004817	XLOC_140078	1.92/0.07	4.754006517	3.51 × 10^−2^
LNC_002885	XLOC_083427	1.47/3.89	2.215701391	3.64 × 10^−2^
LNC_004624	XLOC_133494	5.93/2.28	1.377877824	3.84 × 10^−2^
LNC_001782	XLOC_050277	1.04/3.95	−1.923392878	3.92 × 10^−2^
LNC_005777	XLOC_169074	5.09/5.98	−0.231523482	4.02 × 10^−2^
LNC_003630	XLOC_103678	3.66/2.05	0.833707374	4.28 × 10^−2^
LNC_004460	XLOC_127505	1.58/6.24	−1.978854313	4.74 × 10^−2^
LNC_004835	XLOC_140490	4.44/1.61	1.465355203	4.98 × 10^−2^

Note: FPKM: fragments per kilo base of exon per million fragments mapped; HSHS: heat stressed Hu sheep; WHSHS: Hu sheep without heat-stress.

**Table 6:** Gene ontology (GO) enrichment analysis of the protein-coding genes co-located with the differentially expressed cis-acting lncRNAs (GO level >3).

GO Term: Biological Process	Observed *	*p*-Value
GO:0072593~reactive oxygen species metabolic process	4	1.07 × 10^−9^
GO:0051239~regulation of multicellular organismal process	4	9.47 × 10^−9^
GO:0045927~positive regulation of growth	4	9.47 × 10^−9^
GO:0010897~negative regulation of triglyceride catabolic process	4	3.67 × 10^−8^
GO:0090209~negative regulation of triglyceride metabolic process	4	1.74 × 10^−7^
GO:2000377~regulation of reactive oxygen species metabolic process	4	1.74 × 10^−7^
GO:0010896~regulation of triglyceride catabolic process	4	2.83 × 10^−7^
GO:0048583~regulation of response to stimulus	4	2.83 × 10^−7^
GO:0060192~negative regulation of lipase activity	4	4.30 × 10^−7^
GO:0016043~cellular component organization	8	7.78 × 10^−3^

Note: * Number of genes targeted by the differentially expressed cis-acting lncRNAs; GO level > 3: each GO term contained more than three genes targeted by the differentially expressed cis-acting lncRNAs identified in our study.

## Supplementary Materials

The following are available online at https://www.mdpi.com/2073-4425/10/5/395/s1. Additional file 1: The 520 differentially expressed mRNAs in the liver of Hu sheep with and without heat stress (XLS). Additional file 2: Gene ontology (GO) enrichment analysis of the differentially expressed mRNAs (XLS). Additional file 3: KEGG pathway enrichment analysis of differentially expressed mRNAs (XLS). Additional file 4: Details of the differentially expressed lncRNAs (XLS). Additional file 5: Sequence of the differentially expressed lncRNAs (DOC). Additional file 6: Protein-coding genes detected 100-kb upstream and downstream of the differentially expressed lncRNAs (XLS). Additional file 7: Gene ontology (GO) enrichment analysis of protein-coding genes co-located with the differentially expressed lncRNAs (XLS).
