# Supplementary material for: Heat Stress-Responsive Transcriptome Analysis in the Liver Tissue of Hu Sheep
Source: Genes (Basel). 2019 May 22;10(5):395. doi: 10.3390/genes10050395 (PMC6562622; doi:10.3390/genes10050395)
Supplement: Supplementary file 1 [file genes-10-00395-s001.zip › 2019.05.06-Additional Files/Additional file 5.docx]

>LNC_005888

CTGGATTCCAGCGAAGTGGATCCTGGGAGGTTGGTAGGACCTCTGAACTGGAGCCTCCCATCTCCTGACCTCTCCAGAATTCTGGTGGCCATTTTCCTGTCACTGTTTCTTGAGAAGACTGTCCTTTCCTCTTCTCTCGTTCCTGGTTTGATTTTCATGAACTGACCGCATACATGCGGGTTCCTTCCTGGACTCCCTATTCTTTCCTGGAGTCCACGTGTCTGAGTTCCTGCCAGTTCCTCACTGTGGTGGTCACTACAGCTCCGTGATGTAGTTTGAAGAGGGATAGGAAGATGCCTCCAGCTGTGTTCCTCTTTCTCAGAGTCTTTTTGGCCGTTTAAGGTCCTGTTCAACAGTAGCAGCCTAATATTAGGAATATTTCTATTTCTGTGAAAAATGCCATTGAATATTATTTTGTGATTGCAGTCCGTGTATTTGTTGATGACTTTGGTTGGAATGGACCTTTTAATTCCTCATTGTTCCAGTGCATGAGCGTGGAATAATTTTCTGTGTGTTTGTATTTTCTTTGGTTTCTTTATCAGTGCCTTCTAGTTTTCTGACTACTGGTTTTTTATGTTCGTGATTAAGTTTGTTGCTAGATGTTTTATTTAAGTGGGATTTTCTTAATATTTTTTTTCTGATTGTTATTAGTGTATAGAAATGCAAGTGATTTTTGGACTGTAAAGGGTAGTATTTTATGATTGTAAATTAATCCAAAATGATTCAG

>LNC_004811

CTGTATACTCAAAGCTATTGTTTTTCCAGTAATCATATATGGATGAATATGAGAGTTGGACCGTAAAGAAGGATAAACGCCAAAGAATTGATGCTTCAGAACTGTGGAGCTATAGTCCCTTGGACTGCAAGGAGATTCAACCAGTCAGTCCTAAAGGAAATCAATCCTGAATATTCACTGGAAGGACCAATGCTGAAGCTGGGGCTCCAATACTTTGGCCACTTAATGGGAAGAGCCAACTAATTGGAAAAGACCCTGATGCTGGGAAAGAGTGAGGGCAAAAGAGAAGAGGGCAGCAGATGATGAGATGGTTGAATGGCATCACTGACTCCATGGACATGAATTTAAGCAAACTGCGGGAGATGGTGAAGGACAAGGAAGCCTGGCATGCTGCAGTCCATGTGGTTGTAAAGAGTCAGACATAGCTTAGGACTGAACAACGACAACAACAAAAAGAAAAAATAGAAAACAATGGAAATTTTTAAATGGATAAAAAGTAATGAATATGAAAATGCATCATGTCAAAATTTCTGGAATGCACATAAAAAGTTGCTGAGAGGGAAATTTATTGCAATATATGCTTACAAAAACCTTCCCTGGTGGCTCAGTGGTAAAGAATCTACCTGCCAATGTAGAAGACTCAGGTTCGATCCCTGAGTCAGGAAAACTGACTGGAGAAGAAAGCAGCAACCCACTCCAGTATTCTTGCCTGGGAAATCCCATGAACAGAGGAGCCTGGTGGGCTACAGTCCATGGAGTCGAAAAGAGCTGGACCCAACTTAGTGACTAAACAACTACAACAATGTACAAGAAATGTCTGAAAACAGTAATCTGAGCTTCTTTCTCAAGAGACACAAAAGAGAAAATCTAAATAAACTCTGTGATTGGAATGAAGGTAATAATGAAAAATGACAAATCAATGACTTTTAAAACAAATAGAGAAAAAGAAAAACTAAAGCATTGAGAGGAAGTAAAATTCCTGATTAAATAGAGAATACCATCTTTATCAATGATCAAAACACTTTAAATCAGCTGTACCCCAATACAAAATAAAAAGTTTTTAAAATGATTTAAAATAAATGCAATAATTTGTCTTTTAAAATTTTTAATGAGCTTATTTTCATCCAAAGGAAGTGATTTGGTGTAATAAAGCTGGAAACAGTATAATTTCTAGAAGTTAGCAATATTCTAGATATTAAGAAAAGGAATTTTGTCAAAGTAAAACAGGAATTTCTATTGAGGGATAAAACATTAGAGAGGATATGGGAAAAAAGTAATATACCAAAATATTTCTCAAATGTTCATTATTGGAGGAAAAACTGAACAAACAGAATGTTGAGGCAGAGGTTCCAGAAAAAGCCCATTTTATTTGGAAACTTGGGAGTTGATTAAGTTTCCATTTTTAAGTAAGTGGCAAAATTATGATTTGAAAGAATGAAG

>LNC_003332

GTGAAACTGCAGACACAACCCTCTGAGGGAGGCGGTGGTTTTGTTTCTGGTTGTTCTATAATTGGTTTTGGGATTTGGAGCAAGAGAAGCCACTAATACAAACACTGAAACATAATCATTCTGGTTTCTTGGGATATAGGCATAATCGCGTAACAAGATTTTCGTATTCCTGGGGACCAGGAAAACACTTGGCTTATTGCGTGACAATCAGATACTTGGTGAAAGAAGACGCATGATTATCTACTAATATACTAGGATCTGTCGTGATCTGCTGTATATGACAGGATTCAAAGAAGAAGGAGGAAGAGATGGAGAAAGAGAAGATGAACCCCGTCCTCAAGCAGCTGCCACCAAGTAGGGGGCTTGTTGAGACACTGCACGAGCAGGTAAACCAGTGCAGAGAGCAATGTGATGAGTCGCCCTTGGTTGGCTCCACGTTCCACAAAAGAACTTTTTCGTAAGCAGAGGAAGAAGCTTCCTTTCTAAGGGCAGCAAGATCTCTCATGGTGGCCAGTCTCCCTCATCCTTCTGGCTCAGCAAGGTTTGGCTGCCATGTGGAGAGAGGAGCAGTCCAGCTCTGTGGCGTAGTAAGAGGCAAGCAGTCTTCAGATGGATGCTGGCCTCCTGCACGTTGTTGGACTTCAGTGGAAGGAGGCTTGTAATCAGGAGTAACTACTGTACTCTCCATGCCTTCCTGAGCTCCCAGGCAACCGTCTGAAGCTCAGACTCAATTACCACCATTTCTGGGTCAGTGCCACCTGTCCTCCAGGCCCCAGCACCCATGAGCACACAAGCGTCCTGGACCATCAGCCAAGGTGTATTTCAAGGGCGTTTGGAAGCTTTCTCCTCTCTGATGGCCGTTTCAAGCATCCTGCTGGATATTGGGTTCTCCAGGTCCTCACACAGCCTTGCTATAGGCTCTTTGTGACAGCTTTTGTTCCAAGTATTATGCTTACTTAGTCATCTATGCAACTTTATGTGAGGTTAAACAGTGTGAAGATTGGAGTAACCACTCGATAGAAAAGTGTTCAAGGCCCATTTGTTCTTGCTTATGTCGCTCCTGTTCTTGGAGCAGGTTTTGTATTCCTGCAAATTTAAATCAACAAAGTGCCCTTCACTCACACACACTATGTTCATGGACTGAAAACTGCAACTTCAAGGCTGCCGGTTCCACGCTTCTGCAATTTTCTGTGCATCTCTGGGCAGGTGACTGCAATCTTCCATCCACACAGAGGCCTTGCTTCTTACAGGCAGCCAAGGAGGGCAAGGGAGCTTTACAATGACGGTTAGTAAAGATGATCCTAAGTGCAATGCCAATTGCACCAACATAGCTTTGGAAATAAAACTCTGAACACAAAATAGCCCCACTCGGATCCCAGGGAGAGTAGGAAGACACAGATTCCAGTTGAGGAGGGATAATATGGAAGGGCACGTTGCTCTGTACCGCTCAGTGCTTCCTGCTGAGCCCCGGGGTCAGCGGGTCTGCTGGGTGGAGACACCGGCTCTGAGCGTGTCTGTGTGGGTCTGGGATCCAGTTCCCAGATGGAGACAGTGCAGATGGCGCTGGTAGCACATGTGGGTGGGAGGACTCAGCCCCTTGGAGAAGGAGTGAAATCCTATTCCCAGATGGAGAGGTCAAGACTGTGGATATTTTAGGTGCCTTTCAGTAGGAGGACAGCCTTATTCCCAGACCTGATTGGCAGTCCTTTCTGGCATAATGGAACTTACACGTTGGCAAGGATGCTAGCTTGCTTGTCACCTGATTGCCTTACAGAAATAAAACAGAGTGCTCTGCTTGTCTGCGACGTATGACTCAGTGGTGCTGTCTGCAGGAAATGCATGAGAAAAGGATTCATTGGATAATTTTAGAGAAGAAAGCTGGATACAACTCTTCTTTTTGTTAGAAGACAGGAAAGAGAAATATTTAAGATATCTTCAGCTACTGCATAGCACAGAGAAGTAAATACTCAATATCTTGTAATAACCTTTTACGGAAAAGAATCTGAGAAGGAAAATATATATGTATGTATAACTGAATCTCTTCAGTGTACGCTTGAAACTAATGCAACATTGTAAATTATCTATACTTCAACTTTAAAAGAAGCATCTTAGCCATACAGCTGTAGTCAACTGGTAAACCAGTCAGTGGACGTATAATCCCAGTCATTTGGTACTACTGTCCATTTGTATAAAGCTTAACAAAGGGATCAGCAATGGAAAAGATGCCGAATAGTATAACCTTGCCCCATACAATTTATGTTACTTCCTGGCAGTAGAAGCTTATTCAAGAACTATCCAGAATTCTGATTTCCATTGTTTAGTTGGCATTTTGAGCATTTTAGCGAGAAGGACCTAAAGATCGCTCCTCTTAAACATTTGATAAAATGGATCAGAAGTAAAGTGCGCATAATTTCACTTTATCTTTTCTTCGTGAAAACACTGCCACCAGCTGGTTAATAAATGATCTTGACAACTTTTTTTTTTTTTTTAGGTGAGATGAATGAGTCATTTTCCCTTCATTTAAAAAGGGAGCACAATGTCTAGTTTTAGGTATTTTTCTTCTTTTCATAAAAATTATTTGCATCTCATAATGCCTGTTACTCTTCAGTCCTTTGAAGAGAAAAAAATGATCTGGAGAAGGCCTATGCTGGCCACCCCTGGACAAAAGTATAAACATGTTCAAGGACACTGATGCACTTCTGTGCTTATAGCCAGAATCATTGAATTTCTTGATTTCAGTTGTGAATCACACAGGGCCCATCCTCCAGTTCATAGGACAGTTATCCTCACTCCCATAAAAGGAAGGGCGCCTGGTTTAGGGGCCTGACCAGCACAGCAAGTGTCCTCTGGCAGCTTTCAATGCCTTATGTCCTGGGCATGGTCCTTCCTCCTCCACTGAAATGGGATCCGGCTTTCTCTCTGCATTGGTGCATGGTGTACCCCCAAAGAAGCTGTTTCGGCTATAGAACATGTTTAAATGACCTAAGATTTCTTTAGGTATTAAGACGCAGAACAACAGAAAAGGAGGGGAGAACCCTAAATAGCTACATCGGACAAGTATGAAGTAGAATTCATGTCCTGACAAAACTTTATAAAGTTGGAGCCACCATCTCAATGCTTGTGTGCATTCATCCGGGTCACACGTGACCAGGAGCAGGAGCCCCTCACTTGGTTCAGAAACGTCCTTCTTTAGGAGGATGTCCCCACTAGGAGGGTGGGGAGCCTTCGAGGGAGCTCGTGTTTCATGAAGGACACCCTGAATATGCTCCGTGTAGCAGGCACATTCTAAGCTCTTCCTCAGCAGGTAGGAAATGCTGGGCTCCAGGGAAGGGAGTGAAGCCAAGTTCATGTGAGCAGTGAAGGAGGGCCAGGGGTCAATTATGGATCATGCATGGGGCCAGGGTGGGCGGGTGTAAACAAGAGGCAGCGGTCCTTTCTTTAGTCACGGCAACCATAGAGTGGGGAGGGACGACAGCGTTGGCCATGTTGGAGGCTCTCAGCCCTGGAAATGGCTTTTGCTTTGTTTTCGTGAGGTTTGGTTTGATTTTGCCTCCCTTCAGCAGGAGTCCCAAAGCCTGGAGTGTTCCTGACCATACTACTTCAATAGGGTGCTGGTGGATGGGGTCCAATCGTTCAAGAAAATGCAGAATTAACCAAAGCTGAACAATGCATGGTCTTCCCCAAATGTATCCATTTACAAAGTCCCTTTGTACAGAGTGTGGGGCAGGACTGTGTCCAGGGAGTGCCCACTGGGAGACCAGCCATGCCAGCTGCATCTCCTCCATATCCATGCAAGAGTGCATCAGCACAGGGGAACTGAAAACAGGGGCCAGAGTGTGGCCAACGTTTCTAGGAGTCCGCTTCCATGAGGCACATTAGAAAGCTTTGCGATGCATGACGCTTCCCACCTCCCAGCCTGTGGGGTTGATGACGCCTGCCCCACTTTAGGGGTGGGAACTGGGCTCAGAGAAGCCCGCTTAGTGGGCAAGTGGAAGACTGATATTCAGACTCATAAGCTCCTGTCCCTTCCATGCCACATGGCCAGACAGAAGGACAAGGAGACTGGGCTATGACCTCGGAGCTTCTGGAACCTGCTGGTGAGCTTGAACACTTTACTTGGCCTCTCTGAACCTCTGTTTTCTGATGTCTAAACTTAAAGAGTTGGACCCCAATGATCAATTGAAGACTTTAGCTCTCCTCATGTTTCAAGGTTTCCCACAACCGTGATTGTGTGTGTGTGTGTCTGTGTGTGTCCATCCAAAACCACAGGGCACTCAGTAGAGTAACGTGGGGTGGGAGAAGTGTGTAGATTCCCCACTCTTACACCACGATCATATCAACCAAATTTTCACATTAAAACCTAATTTCAGTTGTTTCAATTGAACTCAGATAGAAGCCAGAGAGAGAGAAAGGAACAGAGATGGCATCCATTGAGATCCTTTTCTTTCCAACTAAAGAGGAAAATATCAAGAATTTCCTGGAATAAAAGGATTCATTTTTGCAAAAGGCTTTAATAAGAAAGAGAGAGGAATCATTAAGAATTTAAAATACGAAGATAAAAACTCTCAAAGATTCCAAAGCACCCACATTAAGCTGTAACACCATTTATTAACTGCATGGATTTGTAGAACACTCTACACTTAACTCAGTTGAGCTCACGAGCTTGAAAAGTACTTTTGTAACACCATTACATTATTTAAGGGCAAGTGTTCATTTCAAGGTTTTATAAATGACTGATGGGACTTGAGTTTCATGGTAGAATTAACTCTAAAATAAGGCGCAAAGCAAACAATTTGAGTTTCTCTCAGACTTTCACACTGACCCCCAGAAAAAATTTAAAAAACATTTTTTATTCAAAAAATTTTAATCTCAAAAAGAAAAGTCTTGCAACTTTTTAAAAGGATAATTTGCTTAGAAAACTTTGCTGGGGTACTGCCAACCTTTTCTCTAACACCCAAGCAGCCTGACGCTTTAATAATACCAAGACCTAAAGGAATTCATGGTATTAGTTTTAGCAAGAGATGGTGATTAATTATTAAATGGTTCCAGCTCTTCTCAGGCATCCCAGATCCTCGCTGCCCCACCGTCTGGTCCTCCCAGCAGATTCTCCAAGACATAGAAGGATGCGCTTGGTTCTCTTGGCTCTGCTTGTCCTGAGTCTGGGCGGCTGGAGCTTCTCCTCCACTGTAGTGATCAAATCTCCAAACCAAACACACCCCCAACACTTTCCTGAAGACTGTAGCCTACCAGCTCCTCTGTCCACGGGATTTTCCAGGCAAGAATACTAGAGTGGATGGCCATTTCCTTCTCCAGGGGATCTTCCAGACCCAGGGATAGAACCCAGGTCTCCCGCATTGTAGGCAGATGCTTTACTGTCTGAACCACCAGGAAAGTCCTTCCTGAAGACAAGTTTTCCCAAATTAGATGCTCTTGCCTCCACTTCATCACCCTGGGACCGTGATTTCCAGACACACATCTTGAGCACATCTTACTGTGCTTCAGGTCTGCCAAGTTCCTTCCCCCATGCTACCTGGGGCCCTGACACAGCCCTCCCCTTCCAGGAGACACAACAACCTTGGCTTCACAAAAGGACCTTTTTCCTGAAATCCAGGGCTCTCAGGCTTCCACTCAGTGACCTGAACTTGATTTGAGGGGACAGGATTTAGGTGCTAGGACTGTCCCTGGCTGGGAAATTTTGACAAACAACTCTGCACTTTTACACGTTTTCCACTTGGAAAATTTGTACATACTCCTTTGAGCCATATTTCTTAAAAAAAAAAAAGTTTTGCCCCTCTTTGAAGTGACTCAGCTGGCACAGGAACAGTCTGGAGGTGGAAGAAAATTGGAAAGAGGCTCTTCCTCTGCATTGCCACCCAGGTTAGAGCAGAGCATGGCTGGGGTCAAGGGGGTCCCCAAGAAAACATGAGGAAAACAGAGCTGGATTGCTTCCGAATTCATCTGATGGAAGGTTTTATTTCATGCTGTTTTTACATCATAACAGTAATACACAATTAGTGCTAGCATTGTATTAAAGCCTATTTGAAAATGTTTTCTCAGTGAAGGTAGCAACTCAGTTTAAGTCCAGAAGTTTTGCTATCAATGGGGTAGATTACTGTGAATCAGCTAATTCCCCGTGTGTATATTGAACTCACACAAACTTGCTGTCAGCTCATCCTTGTTGTCTCATTACCTATAACACTTGTTTTAAGACATTCTTCCGGATACATTTGTTGCTAAATTCACTTTATTTTCAGAGGTTAAGAAAATAAAAGAATATACTCAGTGTAGAAAACTGGAAATAGAATGAACTATAAAAGAAAGATAAACCCTATCACCAGAGTCAACTTTCTTTAAATTTTTAGTTTAATTTCACTCCATGTATTTTTTTTAAGAGTGCTTTGAGAATATAGCTTAACATATACAGGTTTTGCATTTTGATATTCTTTTTTCATTTAACATACCATAAGCATTTGCCACTTTATTAAATATTTATCAAAACAGTTAATGTTTGCATGTTATTCCATTATGTTATGCTGTAATTTATTTATCCATTATTTTTTTAGTGCAATGCTCATTTTGTTTTTCAATAAAATGTACCGAGTGCCACTTTGTGTCAAGGGCCATGTGTTGTTAGACATTTTAAATCTTAGCTTTATGAATCTTGTAGTGTTAAGTATATTTTTCATACATCTTTGTGTGTAACTTTAATTAATCTCTTAGGATAAATTTGTTCAAGGGTATTGGTTGGATGGAAAAGGATTAACAGATTTTTTTTGCCAAGTCTTGATTTTAAAAAATTTCAAACCTTCCCCCAGAATAGAAAGTATAGCATAATGAACATTTCACTTAATTTGACCAATTGTATGGACTCTGTGGGAGAGGGCGAGGGTGGGATGATTTGGGAGAACGGCATTGAAACATGTATATTATCATATGTGAAATGAATCGCCAGTCCAGGTTCAATGCATGAGACAGGATGCTCGGGACCGGTGCACTGGGATGACCCGGAGGGATGGGA

>LNC_001340

GCTTCCTCTCCTCCCGCCCCGCCACAAGCCAGCGGAGGAAGAGGAAGGCATGGGTGACACAAAACGGCCAGGGCTTCCTCGATGGCACAGCCTCTTCTGCAAAGGTGTGTGTGCTGCAGCTGCGTTCAGAAGGCAGGATGCTCCAGCCAAGGAAGGAAGTCTCGTCTCTGGTTTAAATTTTCCAGTGCAGCCTGCTTCAGAAATAAATCAAAAAAAAAAGATGCCCTTCCCTCATCTGCTGCAGACAGCTCCCAAACACTCCAGCCCCATGAGATCTGAGCTGAGCTCTGGCCAGGCCTTACCACTTTCCTGTTTTCCTGGTCATGAAGGATTCCAGCCGAGGAAGAGAAACGGTACACGAAGCTTTTTGTCTTCACCCGCTAACTCACAATCACAAGGAAAAGAACTACTCTAGGTGTCTCAACAATGAGATGCGCTGAACCCACCACTCATCCCAGAGCTTGTCCAGGGAAAGTCCAGATGCCCCGGGGTGGGGGGTGCCCCCATGGTCCCCCACACACCCTGTCCCCGGGGCAAAGTCCGGGGTCACCCCAAACACAAGCTTTAGTCACTTTTCATGTGGTGACAGCTTAATAGCTTCCAGCCTCAGATATTAAAATGAAACTGCAAAAATATAATAAATAAAACATCATTTTCATTTATTTCAGATTCCATTTCTTCTTTAAAACATACATAGTGCCAAAGGCAAACTTTGGGCGCTCATGCCGTTCTTGTCCTTGATTTTTGGACACCACTGCTGTGTACCGTCCACAAATGTTGGCACCACTGCCAAGTAACTCTTCTAGAATTGCTTCCCTGCCTGGCAGAGAATTACAACAGCTGATTTCAAACCCA

>LNC_001712

AATTCATGTAGCTTGGGTTATGTTGCGTAAATTTGTATTGTCACAGTTCAGTTGCAGTTTGACTGATAACTTGTCTTTCAAATGAAATCACGTGAATTTGCCTAGGCTTATAATCCCCATAGTCGTTCTGGGGTCCTTTTAGGGAGTGTGTGTGTGTACATTGAGGCAATGCAAACTTAGGTTGCTTAGCTCTTAGTTGACTGGTGCTAGTTAAAAAGCAGTTTTGCTAATACATATTAAAGTGCTTCCTAACCTAACACATCGCTTGCCTTTCTAGTGTTAGTAATGACAATTTGTATTTGCTCTTTAAACATTTAGGAAGGCCTTGGAACTGATCCCCACTCCCATGGAATAAAGTTAGTAGAAAGAACAAGACTGTTTTACTACCTCTTACTCCGAACAAGACAACTTCCTTTATTTTGCATTTTTGGGTGGTGGTTATAGCACTTGTTTTGAGGTTTGACCACCCTCTCAGTTATTTTTATTTTTTCATGTCACTTTAAGTATACCCTGTGGTGTTTTAACTATAATATCATCTTTAATGAAATTCATGCATGTATGTTATGTCTTAAAACATCACTTGTTTTGACTTAATTTGGCTTGTTTTATGCTATCTCTGCTTTATGAGTTTTTTTTAAAACAATTGAATGCTTATTTTTCCACAGCCAAACTCATACTCTTAATAGATTTGCAGTGTTTGTTTTCTCTGTTTAACATATGAATGATTTAAGATATGACATAAGTTACAACTGCTTGGTTTGCTAAGGCTACTCACCCTTCCACCCAGAGCAGCTCAGCAGAGTACAGACATTTTGCCCTTACTGGAAACTATATCCTAAATTCAGTTAACTCCTAATCTTATGTTAGCTGGAATAAAGATATAGTAGAACTTTTGTCCTTTAAAATTGTTTTTCTTACCACTGTTTAGCTTTCACAGGGCTTATTGAACAAATTTCAGAATCGGTGAAACTTTGTGAATAATTTGCAATCTTTTTTAAAACTTTAATAGAATTGGAAAACATATATTTCCTGAAATAAGTTTCACTCTCAACAATTGGTGTAGTTTAGTCCAGCATGTGCCATAACTTTTTATTTAAAATAGAAGTGTCTTATAAAATAGTTAACAGTTGCATGCTTCCTGGCGCAGAAATTCAGTATGCAAATGTTAAAAATTATATAATAGAAGCAGCATATTGAGCTTCCCTGGTGGCTCAGATGGTAAAGAATCTGCCTGTAATGCAGGAGACCCAGGTTCAGTCCCTGGGTTGGGATGATCCCTTGGAGAAGGGGATGGCTACTCACTCTAGTATTCTTGTCTGAAGAATCCCATGGACAGAGAAGCCTGGCGGGCTATAGTCTATGGGGTCGCAGAGTTGAATATGACTGAGAGACTAACAGTTTCACTTTTTTCCCTCATAAGCAGCATATTAGAAGCTATGCAATCCAGTTATGTGTAGTTTTAAATTATTACTGTTTCTGGTTTTATGAAAAAATAAAGCATTAGGTGTGTGTCCTGAGCTTTTCAGATTCGCACATCTCCTGTTGAAACCTTCACTGCTATTTTCATGTTCCATGACATGAGAGAATGAGGTCACTTTTAATGCTATTCAGGGAATGAAAATCAAACTTTCTTGAAGAGATTCTAATATGTCTGTGTTTTGCTTAATGCTTTCCTGTTACATTCCCAGACAGAGGTCTTAAATTTGAGGAAGTAGTTGAGAGACGCCTGGTTGAGATTCTGAACTCCATCTTCCCTCAGAAGGGGATCAATTGCTACTTCTTCCCCAGCTCCCACTTCATTTCTTAATTGGACAGTGGTACTTTATGCAAAAATTACATTTTACAAGCCAAACATATACTGGGAGAAGAATTCTGTTGTATAATAGCAAACACGGACAGTTCTTCCACACACTTAAAAAATGAGGAAATATATCAGGAGTCCTAAAAATGTGACCACAGTTTACATAGAAAAGCCTCAGTAAATAGAAAAACACGCTATACTCTTGGATTTGAAGATTCAAATTTTTTAGTATATCAGTACTAGAAATTAATGGCATCCCAAAATAAAAACTGAAAGGATTAAGAAAAGAAAAAGGCTCTACCAGACAGTTAACTCTGTTCAGGTAAGTATCAGTGTAAGCTCATTTTGTAGATGAATAAAATGAAGCTCAGAAATGAAGAACCTCTCCAAGTCTGTAAAAGCTAGGAAGTAATTGAAGCAAGGTATCACAAGAAGGTGTTTGAAGGCTCAGATGTAGTAATAGCTATTAATGTAATAAGCACTTATATTACTAAGCACTGCTGTTCAGGCAAAGTACTGTATATTTTCCCAATTAGAATAGCATCTAAATATACTTAGAAAGTAAAAATTTGTGAGACTTTTCTAGGGTGGTTCTAGAGCTAATGCAACCATACAGTTAAGTTCATTTCATGTTCTAGCAAGGTTTCCTGCTAATTTCTGTTCTTTGGCCGATACCTTTAATACTCCCCATTCCCCTTGTCCCAGTTACAACACAGTTGCTAGATGTGTAAAAAGCAGTGGGTTCTGTGAAAGTAGGGACCTTGTCCCTGTGCTGTGCTTAGTCGCTCAGTCATGTCCAACTCACTGCAACCCAATGGACCACAGCCTGCTGGGCTCCTCTGTCCATGGGGTTTCTCCAGGCAAGAATACTGGAGTGGGTTGCCATGCCCTCCTCCAGGGGATCTTCCCAACCCAAGGATCGAACCCAGGTCTCCTGCATTGCAGGTGGATTCTTTACCATCTGAGCCACCAGGGAT

>LNC_003730

AGCCAGGCCTGTTTCTCTCGGATCTCACTCTGAGCCCCGTCGAGCTTATTCTCGTGACCGTGGCCATGAAAGTTTCGATGTCCTCTGCCTCCCTCTGATCTTTGCTGCAAACCAGGACGATGTAGGCTGTCTGGCGATGCACCACGTTGCTAACTTGCCTTCAGGTGAATGGATAAAGCAGATGTGGAACATACATACAGTGGAACATTATTCAGCATAAAAAAGGAAAGCCATCTGCAGCAACATGGATGCAACTAGAGGTTATCATACTACGTGAAGTAAGTCAGAAAGAGAGACACAAATGCCACGTGATATCACTGATATGTGAAACCTAAAATATGGTACAAATGAACCTAGCTACAAAACAGAAATAGACTCACAGACTTCGGGAACAGACTTGTAATTGCCAACGGGGAGAGCGAGGGGACAGGTGGATTGGGAGTTTGGGGTTAGCAGATGCAAACTATGATATACAGAATGAATGAGCTACAAGGTTGTACTGTACAGAGCGAGCTTCTGTTCTGTCGATCAGTTGTATCCGACTCTTTGTGATCCTGTGGACTGGTAGCCTGTCATGCTCCTCTCTCCATGGGATTCTCCAGGCAAAAACACGGGAGTGGGCAGCCGTTTCCTCCTCCAGGGGATCTTCCTGACCCAGGGATCAAACCCGCATCTCCTTCATTGGCAGGTAGGTTCTGTATAGCACAGGGAACTCTGTTCAATATCCTGTGATCAACCATAATGGAAAAGAATATTTTTAAAAAAATGTCATATATAATTTCACTTTTAAAAGGAGTAAAACAATTTAATACTATTGCTGCACAATAAGCTCTAAATAAAAATG

>LNC_000765

CAAAAATGTTCCATACATTTGATAAACAGGTTTGCAAAGGAGACCGGAAGCACAGGAGAGAGTCACATCTTTCAACACGAAAGAGTTGCTGGAAGTACAGGTACAGCTGAGCTTCTTGGTTCGGAAAGCAGAAAGGATGCCTGCCGTGGACACAGATGCTGGCAATAGAGACGCCCCTGAGGGGCTTCCCTGCGGGGCTTCCCTGGCCATCCAGCCGTCAAGACCGCCAGCTCCCGACGCAGCGGGCCTGGGTTTGATCCCGGGTCAGGGAACGACATCCTGCATGCCACAGTGAAAGGTTCCACATGTCAAAACGGAGCCCTGGGGTGCTAAAATAAATAAATATTTTTAAAAAGAAATAAGCAAAGAATGAAATTCTTAAAGAAAAAAAAAGAAGGAAGAAATCTGTGGGCTGATCCAGGCAGGCCCCTGTGACTCAGGGAATGGAACACAAGCGGAAAGAGCAGAGGGCATGGGCCTGGCGATGGGCGCGCTTTAGGATCATGGGTCGATGGAAACACTTGAGAGATGGTTGCTCAGCCTCATCCTTGGGGCCAAGGCAGAAGGCCCCTCTCCCCTCGCTACCACAGCCCCCGCCTTCCCAGCCTTGGTCCCCGAGTCTTGTCTCTCTGGACAAGGGAAGCAAACCACAAGGAGAGCAAAAGCCCACAGGTGTTCCAGGAGATTTTGGTCATGAAGGACCGGATATCTGGCTGAAGCATGAGAAGAAAAAGGGGGGGCAATTAAGAAATGGAAAGGCAAAAAAAAAACCCCACAAAAAACCCAGATCCATGTGTTTCCTGAAAGATGTTATTTCCTTAATGCAAATAATCATTTTTAAAAAATTCTAGAGGATGAGATGGTTGGATGACATCACCTAGTAGATGGGCATGAACTTGGGCAGACTTCCGGAGATAGTCAGGGGCAGAGAAGCCTGGCATGCTGCTCTCCATAGGGTCACGAAGAGGAGGACCCGCCTCGGCACCTGAACAACAACAATAGCTCTAGAATCTATGTTCTCTCCTTCTCCTCCCACCAAGAGTACAAAGTCTGAAGGACCCGTGCATGGAGCTGCCCACAGGAATCGCTTTCCCTCCAGGCTGTATCCCGCCCCTGGAATGGGAGTGCACACCCCGGCGTGGTCTCCTGAGGGCCCACTCCTGCACTGACCCCCACACCCCCCAGGCTCCGGGGTCCCTGCTCTTCACTGCTCTTCATTCCCTTCTGCTCTCCTGGACCAGATCACGGAGGTCAAAGCCCAGGGGGTTTGAATGGATAGTACAGATTCTCTGATTTTGTTACCACTTTATCCTGGGATTTTCTATACAAGGTGCTTTTGTGAGATTCTCCAGTCCTCCAGAATTCGTCTCTTTGGAGAGAAACCTCAGCCCCACAAAGGGATGGACGCATCAGAGCAGACGGTGTCCAGAGCTCTTGCCGTGTGGGGCTGGGAGTCACAGGAGCTCCTTTGAAGGGTGGCTCCTCCTTCAGGAAACTCAGACGAGCATCACGAGCTCCTGACCCTTCTTGACCTGAAAGCTGACCCATGCAACTTCACTGTAGCCCACAAAACTGGGGTCACCCCCTTGGAGGTCCTTCTCATCTCCTTTTTGCCTTTTCAAACATCCTCGTTTTTCAACTGTTCTTCCTAGTACATGATTCTTCTCTCCAAGTAAGTTCCAGTCTATTTTTACCAACAAAGCAAATATTTTAAAACTGCCGTTTTGATGGATATGGTACAGTTCACGTATGTCAATCATTAAGCTGTACACCTTAAACATGTACAACTTTTAATCTTCAACTATACTTCACAAATCCAGAAAAA

>LNC_000193

CACTAGCCAGTGCAGGAGACATAAGAGACTCGGGTTTTATCCCCCGGTCAGGAAGATCCCCTGGAAGAGGGCATGGCAACCCATTCCGGTATTCTTGCCTGGAGAATCCCATGGACAGAGGAGCCTGGCGGGCTACAATCCATGTGGTTTTACAGAATCAGACATGACTAAAGCAGAGCAGTAATATTGAAAGAGCCTGGCTCCTGACGACATTCTCGATCGGATGCACCAGCCCTGGGCTGTCTACCTTTGTAGTTGTTGTAATACAGGAAAAAATAAAGAAGGTACTAGTTGTCAGATTTTGTTTTACTTGCAGTTTAAGTCTTTCTAATACTCTAATTATTCCTCCTTTGCTCAGATACCCACCCTGGAAGCAATCAACAATAGCTAAGGGTAGAAGATAGGATCATGTTAAGACACAGGGGACCTCCCTCTATAATTAGTGTTGGAGAGAATGGGTGGTGAGTGTGGGATGTTATTTCTAGAAAATGACAATTAAATAGAAAGGAAAATGATATTGTAAATGCCTACTTTAGAGAGAAGTTTTTGAAGATGATTCCAGCAATTGAGCCAGTGTGGATCTAAAATTAACAAAAATATGAAATTCAGAAAGAGAGGAGATAGGAGGTAATAACTTTGATCCAGACAGTTATTGAAAGCGTTATTTCCTCCAGCTTAGCTGACGCTCTCCAGATGGTCATTTGCGTGAATGCAGGAGTTCCACTTCTTTCCACCCACCCCTTCACTATACTATGTTCTCAGCATCCATTCTAGATGGCTTAAAAATATCATGGGTCAACTGAAAAGAGGAAGCAGGGGGGTCATTTTGGAAAATGTTAGGGGAAAAGTGTGATATATGCAAGTGTCCTTTCTGTTAGGCAGTCAAGGAACAATGGGCATTAGATTGCTACTGAGTAATCTTAATGAGCAGACAGAAATAAGAATAACAATAACATTGCTCCTGGGTACCTGCCTCCATGATAACTTCACTTGCAACTGGTGGTTTTTGCATAAGAGATGAAAGCTGCTCCTCCCATTCACATCATGGAGAATTCATCTCAGGCGGTGGATGGACTAGAATATTGTCTTGGAAAAGGAGAGGGTAACAGGCAGGAAGGCTAGGGGCCCCGAACAGAGGAACTAGGCTGCAAGTGTCAGATGTTTTTTGTCTCTCTCTTAAGTGGCAGGAGGGAAAAAGCTACAAGTGTCAGATTTTTTTTCCCTTTTCTATACAAAATTAAAAGGAGGTTTCTTTTAGAATTCTGTGTTGACATGATGATACCTGGTTCCATCTGAACTTAACTTTTCTCAAACCTTGAGCTAACCGATGTCTTTTTCTTATGGAAATATTCTTCTTGAGCTATGTTAATGAATTATGTATTTACCCTAGACTCTGTCTTGCTTCAAGTCGGTTCTGCCTAAGACTCAGAACCTATAATGTTTCAACAAACCAGTGCGTTTTACCCATGGAAATGTTCTCTTAAGCTGTGTTAATGAGCCTATGTATTTACTTGGAAACCTGTCTTTCTTCAAGACTCATGTCAATCATTTTATGGCCCAGGATGACACCTTGTGCCAGTGTTATCTCAAAATGAATGTTGTGGGGTGCGGGGCCTGGTGCCACTCTCTGAGTTCTGAGACATTTCCTTTCTCTAATTAGCAGCCTGCTGATAGGTATGTAACTTCTTGCTAAAAACTAGCAATGGGGCACTCTTTCTGCCCCCTCTGATGTCTGTGTCAGAAGCTTTCTTTCTTTTACACTTTAATAAAACTTTATGACACAAAAACTCTGAGTGATCAAGCCTTGTCACTGGCCCTGGATCGAATTCCTCTCCTCTGGAGGCCAAGAATCCAGGCATCTTTCATGGCTCAGCAGCAATCTTTCAGAAAAATATACATTTTAAAGCCTTTTGACGTAAACCCATAAACCCAAACATTTTAGTAAGTTTAGGAGCCAAGAATCAGTTTCCTTTGTTCTCTGCATCTATCTAGAGTGAGCTTTTCTCCCCTATCTGTTTAGCTAGAACCAGGAGTTTCTTCTAGAAGTGATGGGGTTGAATGAATGTAGAGGGAGAGAGAAGCACATTTGAAACACTTCCCTCATTACAGACACATTGAACTTAAGATTGTGGTGGTGTGTGTAAACTGAAATGTGGGTCTAGAGAGGCATAAGGACTGAAAGGGTAGATTTATGAGATACCCATAGCTGAAATGATGTAAAGTTCACAGAATAACTTTGAAACAAAGGGATAAAGGGCTCAAAGTTAGAAGACAGGGCACCAGACACATTTAGGGACAAAAGAAACAAGAAGTCTTATGTCCCAGAAGCATGAGATGAGGAGCCAAGGAGATAAGTGTGGCCAGTGGATAGAAAGCTGAACTTTCTGATTGGAGAGCTTAGTGCTGAGTACTGGATACTAGAAATTATGAAATCACTGGATGCCTTTGTAACAAGAGGTTCAGAAAACCAGAGGAGGGCAGGTAATTTAAAGGAATCTGTAGAACAATAATAGCTTGGGTAGGCACTTGTAAGACTGATCTTTTGTCTGAGTTGGAAGACTTGAGCATGTTTTTTGTTTTTTGCTGAGAAGAGTGAGGCAACAGAGAGAACCAAAAAGGCAAGAATAGGCACCATTAGGAGACCCTGAGGCCAGACCCAAGGTCTAATTCCTTTGCTCCTTTTCCCAAATTGAACCATTCAACCTCCAGTAAGAGAGCACAAAGGTCAAGGGGAATAGGAATGAGGACTGAGGGAAAAGATTAAATACTTTTGGTGCTAATACATGTAGCTTCAAGGGATCAGGCTTTCTTGCTCCATGTATTTGACCCCCCTCTCACCCTCATTCTCACTAGTTCTGAGGCAGCCTGTCCTAGTCTCCACTGTAATTCAAGAGGTTATGAGAACTTTGTGGGATAACTATTCTCAGTGATACTGAGTCCTGGTCACTCATTCTTCTATAGACATTTATTTAGCACCTTTTATGTGCTACATTGAAAACAACCTCATAAAAGAAAGAACCACTGTTACTACCCTCACGTGATGGATCAAGAAACTGAAGCTTAGGTTTAGACATAGAGCAAGCGTGGAGTTTGTTTCTAATGTTGTTTTAACATCACTTTACTATCTGATTCAGAGCCTTTCTACTAAAACAATGAGATCAATATTTTCTAAATCCTTACATCTTGCTAGAAACTTCAGAGTACACAAAGGAGCTAATACCCTCTCCCACTGTCCCAGAAAGAAATTCCATTTGAGCCACCAGACAATTTCCTTAAATGAAATATCACACAGTCAGTACCCATTTTGCATAAAAATGACCATGTGAACAGAAGCTAAACCAATCAATCTTAATAATCAATGTAAAAAAAGAACTGACTGTTCTGTAATCTTTAAACTATTTTGTCAAAACATTAAAGATTTGGTTACTGTAAGTTATAAATAAAAAAATATCAAAACTTGTAAGGCTCGGAGAACCGTGCTCTGAAAAGGACACTCAGGGACAGCCTCTAGTGGACTGACAAAGTTTATTACCCAGTTGTGTAGTTTTATAATTTTCAGGGAATAGAGCATAAGGCTGACTTGCACGTAGCCATTCCAGATAAACATTAAAGCATTCAAGCATAAAATACAGTTCCTACCGCCCAAGCCATAACATAGCTTTGGCGAAACTCTAAAGGGAGTCTTATCATGAAACAACAGTCCTTGCTCTCAGAAACAGGTGGTCAGAAGGAAGCCTTCGAACGCCTCAAGGCCGGACGTATTGACCCTTTGTCATCCGTTCCCAGCCTTGGGCACTCAGTGAACGCTCATAACCCTTTGTTATGCTCGTCCCAGCTTCCAACCTTCGTCAGGAGTGGAGCAAATATATTTTCAGTATTTGCTTAAAGCCTTCCAGCTTCTCCACAAAAACTAATATTTACTTAGTTCCCTTCAATTTAAAACATTAGAAACATTCGGAAGTAAAGTGTT

>LNC_003439

CTTGTTTACTTTAAACCCCTGGATCCCATAAAGGGAGTGGGGAAAGGCTTAAAACCCACAGACGCTCAGGGCTGGCGCCCACAGCTGCCCCCACACAGCCTGCAGAAGGCACATCCCACTGGCAGAGAAGGGGCCCGGGGCAGAGAGACGTCCCCGAGGACTGGAAGGTAAGTGCCTCCCACACACTTGCCAGCACCCTCCAGCGCCTCTAGGCTGTCCCTTTGCGGGGGAGCGCTGACTTTGAGAAGTGGGGGTGAGGTGCGACCCAGGTGTTTAGAAAGACCCCCTGAGCACTGTGTCCTGTTACACACAGCACATTTTAATTCATCTGCCATGATTGTTCTGTGTCTTCACAAAGCCTAAGAAAACCAGAGACCTTCCTAAAGATGCTTCTCCCACCCAGTGGAGGGAAAGGAGAAGATACTTCCCAGGTGGCTCTAGTGGTAAAGAGCCCGCCTGTCACTGCAGGAGACGTGAGAGACGCGGGTCGGACCCCAGGGGTGGGAAGATTCCCTGGAGGGGGCCGAGGCATCCCACTCCAGGATTCCTGCCTGGAGAATCCCATGGACAGAGGAGCCTGGCAGGCTACTGTTTATGGGGTCACGAAGAGTTGGACACGACTGAAGCAACTCTGCACACGTGGAGGGGTCGTGGACTTTTATTTCTGGCCTTGGAGGGGCTATACATGAAGCCACTTTCTATTTTATGATGGTCTGATCCAGCAGTAACGTAGAGGCCTTTGAACAAGGTTCTACCAGTGGGATACTAGCATTAGGACATTTATTTGACCAGAAACCCTCCCCTTTCCCTTCTTTCAGAGCAAGCATGACCGCAAGGCTCCCCATATTTTGTTCTTCTAAAACTCCAATGTTGGAGTTCTTCCTCTGTCCAACTAGGATTGTTCCCTGGACAGCACTGGGATTAGCTGGGCACCTGCATAGAAATGACACTGGTTCCTAGGAATCCCCAGACCTCAGCAGTTCTCTTTAAAAAGAGTCTACTTTGGGAATGCATGTAAGAATTAAAGATTTTAAAATTTAAAAAATAAAAAACTAAAATTAAAAAAAATAAAAAAAAATAAAAAGAGTCTACTGAGGGACTTCCCTGGTGGCCCAGCGGCCAGGACTCCAAGCTCCCAGTACAGGGGACTAGGGATCGATCCCTGGTCAGGGAACTAGATCCCACATGCTGCCCCTGAGACCCAGTGCAGCCAAAAAATGTTAAAAAAAAAAGTCTACCGAGACCCCCAACTTGGTTGAGAATATGCCCAGTGATTTTCTCTTCACCTCTGGTGATAGCAGGCATAATAGATACATGCATAAGCTTTCCAGATTCCCCTGATTTCAGGCAGGAGAGGGCAGGAGGAGAAGGGTGACAGACGACCAGATGCTTGGAGGGTATCACCAACCCAGTGAATGTGAGTTTCAGCAAACCCTGGGAGATGGTGAAGGACAGGGAAGCCTGGCGTGCTGCAGTCCACGGGGTCGCAAAGAGTCGGACGTGACTGAACAACTGAACAAAAGGTAGATTTCAGTGAAAAGAGAAATCCTTCCTTGCCCTCTATTGAAAAGAACGTTCTATTTTTAAATTTCCATGTGCTTTCAGAATGACAAGACTCAGGCATATGTTATTGACTTCTTTTGTCTTCTAGACTATAATAGCCTACAAGACTCAAATTGATGTGTTTAATACATCTATTTGAAATGACAGGCAATGCGAATGTGGGATTTTAAAAAGCGCATCTCCGTGTCTCACTTGCTTTTTAAGGATCTCTTTTAGGTGCTGAAGGGGTAGGTTTGCGACAAACAGGCACGGCGTGGTCCCCAAAGCACTGTTTTCTCAGCCCTCTGGAGTTGGCCCTGGAAGGTTCTCAGACCCTAGGAAATGACCTGCGAAAGCAGGCCAGGTTGCTCATGCCCCGACCTGACCCAAGGAAGCACAGAGAGCTTTGTGATATTCTGGGTACTTGCAAGTCCAAGACATCTTTCAGGGAGCTGGGATGCACTCAGAATACTTACAGACTGGTGAATTCACCAGAGGATCAAGCTTTGTGAAGGCCGCCTTCTCAACAAACTCCTCCCCTTCCTGCCTGTTCCCCGCTTAGGAAAAGAAACCCTCGACTTTAAAAGAGGAGCAAAATCCAGTCTCTCCCGATTTTCATATCCTCGAGCTCAATAGGAAAACGGCAATCTGCATTGACCACACCAACGAGAAACATCTATAGATTCTGTCCAGGTTGGGAAGGCCAGGGAGGGGAGCCAAAGACACGGCTCAGAAATTTCATTTTAATGAACAGCCGTTTAGGAATGCCTGGGATTCAGCTTCTCTCAGGAAATCTCAGGAAGTAAGGCTCAAGGAGAGATGGTGGGAGATTTAGAGAGCTGAGATCAAAGGAGAGAAATTTAACAAGATCCCCCAAGGCCTTTACTAGCTGTGAAAAAACTGCTGGCATGCGGATGCATCACCCTGAATTAATTTCCAGCCCCTTTGCCTTTAAGGATTCATTTTTTTCACAGGAAATAGCAGGTGTAATGATTTCCCAACCAGTTTATAAACTACTTTCAGTTTTTCATTAACTGGTGCTATTTAAGTTCAGGGGTTAAATTTGAAAATATGAAAGTTTTTTTTAAACCTACAGAATGAGAAGTGACTACGTAAATTTTAAAAACTATATCAAAGTAGATCCTGGCATTAACCACTTGCAATTTTTTTTTTATCGGTGACATATGATTGGATGGCATTTGAAAAAAAATGAGAACATTCGACTTGCCCATTTAAACTACTTTGCAGGTGGACAGAATTCAACCACTCCTGCCAAAGCATTTAAAACTGTTCACAAGGCCAAGAACATAATAGGCACTCAATAAAGGTGTTTGATTGATGCCATAATACCTAAAACAGTTATACTGTATAATAAAGCGTTTTGCTAATAATTGCTAGGAAACTAGCCTGCAGCTCCATCTCTCAGCGAAGTGCTAACTTCTCATGCTTGTTGGCAAGCAGAGTAGGAAGAGGGTGAATTCTAACCTCCCGATTTTCAGGGGAATGTTTTGCAATAAGGAATTTTTATTAGGGAATCCACGCTCCCCCCCAAAAACGGTTCTTAAAGATGTTTACATTCTAATTATTTTTCTCATTACAAAAGCATTACATTCATTGTAGAATTAATAAATGCAGAGACCCAAAAGGAGAAACAAAACCGCACTGTAATTCCACCACTCTGGCATAGCCAGGGTTAATAGTTGATATGTTTGTCCCAGGCATATATACCAGTTTTCACAAAAAGGGCATTTGAAATGGTTTTACAAACTACTTCTTCACTGAATCTCTTTCCAAGTCAACAACCATTTTTCTATAACATCACCTTAAATGACTAGTATGTATTGTAAATGTCATACTTCTCTTATAATTGGACAGTTGCTTGCAGTTTTTATTGTTATAAACAGTGCTCCTGTGGACAACCTGACAGGTAAACCTTTCTACAAATGGGTAAAAAAGAAAATCTTTTCTACAAATTTGAGGACTTTTTCCTGGCCACGAATTTCCTGAAGAAATCGAGGTTTATTTAGAGGAGGTAACACCATTAACACATTCACTGCCTCCTCTCTTTTCCCTGTTTACGCTTCTACCTTCTGTCTTTCGCAGGTTCACACGGGGCTCACCTGGAGATGGGGAGTAAGGTCAGACTTTGCTGGGAGGCTCTGAGAAGTGAAATGACCTCAGGAGTCTATCTGGGAGAAAACCCTCCAAGCGTGTCCTACCAGGACCTTTGTCACTAGTGAATGATTCTTTCCAACCAAGCTAAGATTTCTGCAGCCTCTCAGCATGATTGGTACATCCCTGGAGTGACCAGGAAGAGGGGTTTCTGTTCTATGTCCTTCAGGCAAAGACAAAGCGGATGAACCTGGCTTAATCATCAGGAGAGGGGAAAAACCAGAGATGGTTAATCACATGAGCAGCTGCAGCCAGCATGCCAGGGCTTTGCGGTAGCAATCGGGGTCAGCGGGAGCTCTAGAGCCGGGCTGCAGTGGGAATCCTGGGAACAGTGCCCAGGGGAAGAATTCCCCCTGAAGAGAGCAGACCTCACATGGGCTGGGTGGGACTCTGCTAATGCTCAGCACCCTGTAAAGGTGATCCGGGGTCTCAGACAGGAACCAAGGGAGGTCAGTCCTTAACGGAAAGATGAGCACATGAACACAGTAAAAAAAAAAAAAAAAACGAGAAAAAAGTGCTGATGTTGTACACCTAAAATGAATATGTTTTACATTTCGATAAAACTAGAAAGGAAAAAAAGAGGCAGGGATCAACATTCTGACCCTGTGGGAGCTGAGTGACTTTCGTGGTATGCTCACAGCCACACAGCCCAGGCTGAAGAGGGCTGTTGCAAGGCCTGCCTGTGAATAAGGGCCATTTTTACTGAAAAAAAAATATGCAGAAAGGTTCATTTTCCAGTTATTACAAATGGGTTGATTTTATGCCCAAAGCGAGGCTCTGGTTAAAAACTCAGTTATCCTGGGGGAAAGAGTAAGCTGGCCTCCACCCTTTTCCATTTCCAGCATTTGGGGGAAAGAAAGGATTAGATAAAATGCTTCCTTTCCTCTCACGTGCCATTGTGGGCAGGAGCGCATATTTCAGGCAGAAACCGTTTTCAACAGGAAATCAAGTAAACCAGGAGGGATTTATTCACAGGAGGCCCCCTTTGGCTAGAAAAGGAGTTACTAACAACAATGGAATAGTTCTCTTTCCACGTTCCTCCTCTCCCCCCACCACCATCTCCCACTCGCTACTGTGACGGGAAACCACTGCGGTGAGTGAGAGCCGACATCCGAGCCCCTGTGAACGCCTCTGTATCCAACAGCCACACACAGAGTGGATCACAGCTTGTCTAAACGCTAGGTCTGTTCTCAGACTGCAAGGTTGCCCCCGGTTACTGATGATGGACTCGGGTAGATGGTGAGGAAGAGAGGCAGGGGGGAGCCAGGACTCCAGTCGCATTATTACCATTGATGATGTTGCCTTCCACTAAGTACTTGCTTTCTAAAATCAGGTCCCACTAGGGATTCTTCCTCTCTCTTCGCCCTTTCAGAAAATTGATGCAAACAGATTTCCCATCCATTTCCGCAGGAAGTCTTCCTTCCCGGTGCGGGTGGTCGCAGATCACAGGCAACAGATCCCTTTATTAGCGTGTCTGTTCCACAAGTCCAACATCTTTATTTCTCTCCAATGCTCAAGATGTTCCCGACCTGTTATCCCTCTTTTTTGCTTTGTTCAGGTCTCTCCATGAAACCGACAAGATTCTCTGATGATGCTCAGAGACTCTGGTGGAAGACGGAGGTGAAATCAACACTTCCACGGCTACCCATTTCCCTTAGCAGAGCTGTGGAGTGTGACAATGGTGTTTGTGTCCAAACTATCAAACGCCATTATCACACTAAATAGCTACTGTTAGGCAATCCTTCCTACTCCCTGAAGGTACTGACATCTGCTTGGTGTGGGCAGGAAAGTTCATCTCAGAGCAGCGCTCCCAGTCCTGTGTCCTTGCTGAGGAAATTCTGGTCCTTTGTGCTCATAACCAATATGTCTTGTGAATGTTGCCTCTTTTCATGCTGTTGTTTTAAATAATTTAATTGATTTTGAGTCTTTGTGTTTTCCCAAGTCAATAAAACAATTGCAGCGTCCCCCAGAGCACCAGGTTAGTCTCGTGGTAATTGTTCAACCCATGCTTACTGAGCTGAAAGGGAGAGGAAAATGGTAGATGCAGAAAACTAAAATTGGTTTAAATCTAGGGTTTGTAAACCACATGCTTATAGGTATGAGGAATGAAGGTATGAGGATCTTCTACTTGTCATCAGGAAGACAGCTTTCCAATAAACTTGTACATGGCTTAAATGAGCTTTCAGAAATTCTAGAACATGTTTGATTCCTACTGACTTTGCTCAGTCACCAGGCTGACGACTGAATTTGTATGCTAGGTCCTTCTTCCTTATGTTTCAATGAGTTTCCATCAGGAAATTACATAAACTACGTATGGGCTGTCATAGCTAATCTGAGCATTTTAAACCCGGTGTCGCACCTGGGACACTTGGCCAACCGGGTGGCCTGGTCTGAAACATGTTCTTTCTGTGAGAAGCATTTAAAGCAAAAAGTAGGCTCTGTATATGCCGGGCTAAAACTTCTCTCCTGTGACTTGGCTAAATGAACACATATATGTTTAATCAGGGGCTTCCCTAGTGACTCAGTGGTAAAGAATCTGCCTGCAATATAGATGAGGGTTTGATCCCTGAGTCGGGAAGAACCCCTGGAGAAGGAAATGGCAACCCACTCCAGTGCCAGGCCCTGGGAAATCCTACAAACAGAAGAGGCTGGTGAGCTAGAGTCCATGGGGTCGCAAGAGTCAGATTCAACTTAGCAACGAAGCCACCCCCAGGTTTAGCCAAGTTGAAAGGGGCTGGAGTGACCATCAGAGGCCAGTTCTGATTTAGTTATTTATTTACCCCTCATTCATCAAGTAAACGTATTGAGAGGCTACCGTGGGCCAGGCACTGCCCTAGGCACTGGGGAGGCTTTTAAATTAATGGGCTCACGATCTGTCTGAGACTATTCTAATTGTTTCAGTTACGTTTTGGGAGGGAATCAAATGAACAAAACTTTGCCAAAGAGTTCCAGGTTCCTTGCTGGAAAGGACCACTTAAGTCCAGTACACATGCCTGTGTACACAGCACAGGTCCCGAGGGACTTCCATCCGGAGAAGAGGAGGGTCCTTTCCTGTCTGACCCCCAGCTCCTTCCCCTCCCATTTCCTGCCTCCTGACATTGTCCACACTCAAGAGGGGAGCCAGACCCCCCGAAATGATTCACACACTGATACCTTTTTTGTGTGTTTTTCTGGAGAGTAGACAGAGAGCGCGACTTTACAGGAGTGCTCAGTATTTCACTTGACCAGACAACGCAGAACAAAGCTGCCAGGATTGGAATCCACAGAGACTCGGTATCCACTGGGACCGGGCGTGGTTCTGCAATGGTCTGGTCACACGAGTACAGCTCACGCGGGTCTCCTGAATCTAAGATTCTGTCTCTTGGCTATTATCCTTCCTTGACCTGGTGGATATTCCACACAGCCACATGCTTCACCACTGAGGCTGCGTGGAAGCTGGGTTAACATCCTGGTATTGGGTCAGTATTCTCAGCCCCTGTCTGCTTTCTTGGGGATCTGTGGGAAGCCCCTAGGATAGGGGAGGGGTAAGTCATAGGACGATCTTTCCCTGTGGGGTCTCCAGATGCAGAGAAAGCCAAATATCACGAAACCCTGAAACCAGGAAGTCAAAGATAAGGGTAGAGAAGGAACATCTAAGCAGTAGTTTGGGATCAGGAAGCAGGTGGCCAAACTATTCAGACAAAGACCAGCAAACAGGGACCCAGCCAACCACGAGGCACTGCCAGCCCCAGAGTGCGCTCACAGGCTCAGCAACAATTCAGGTTGGCCTCATGCGTTCCTCCCTCCAGGCGAGAAGACTGGAGTGGGTTGTCATTTCCTCCTCCAGGGGATCTTCCCCATCCAGGGATGGAACCCGTGTCTCTCATGCGTGTTGGCAGAATGCCAGTGCGAAGACCTCCGACTAAAGAGCTCAGGGGAGCGTCCCGCGTTCCCAGCTCTCTGAGCCCGGGACTGCTCTCTTCTTGCAATACTGCCCCCTACAGGCAGGACTTGTCCCTGCCCATCACAACCGCTGAGGTTGGCAAGGATTTCATTACCCGAGTATTCCAAATTAATTCATCCGGAGCTGCCTCAGAAGGGAGTCACCTGCTCCATCCGCATAACTATTTCCTGGACCCCGTCTCCATAAGCTGTGGGCACAACTTCTGCCGTCCCTGTCTGAGCCTTTGCTGGCAGGCCAGAGCTGACCACAACCCCAGCTCTGAGGAGCAGGTCTGTGTAACATACTAGGAAGCCAAAGGCCTCTTCTGTGAGGCTGACCAGACCCTGCTCTGAACGCCCCGAGCACGCAGCTCACAGCCACAGACCGATACACAGGGCTGCCGAGGAATCCCGGGAGAAACTTCTGGAGAGAATGGGCTCTTTACAGAAAATGAGAGAAGAAATGCAAATTCTGAACCAGGAAGCTGGAAAAATTCAGTCATTTAAGGAATATGTAGCCTTAAGGAAGCTCAGGATTACAGCTTGATACCAGAGGATATACATTTTGCTCCAGGAGGAGCAGCAGCTGCCTCTGGAGGCCCTGAAGAAAGAAGCTAAGGACATTTGTCTGTAACTCAAGGAGAGTGTGTTCAGGACGACTCAGAGAGAGAGGCTGAAAGAAGTGTACAGAGAGCTAATGGGATGTGCCGCAAGCTGGACACGGAGCTGCTCCAGGGCTTGGAAAATGCGCTGGAAACTGACGTGGCACGGATGCAGAAGCCGCAGCCCGTGAACCCAGAGCTGCCTTCCTGGCCCATCTCTGCATTCCTGCACGTGCCGAACACCTTCAGTGTGAATAATGTTCTGAGTCGGACAGCGACCGTTCACCATGCGATCCTCGATGATGCAAGTGTGATGTCTGAGGATGACCACCCCGCGTGTCCAGACAGTCCCAGGGGCGGGGGGCGGCGTGTGCCATGTCCTGGGGGGGCTTGGGCCTTCACCTCCGTAAGGCATTACTGGGAGCTGGACGTGGCACAGTCCTCAGCTGGGTTCTGCGGGTCTGTAAAAGCATCTTGACAAGTGATACCAGCATCAGTATTGGTGCTGAAGAAGCATTTTTTCTATATTCTACAAAGGTATGTTCTGTCCACCACCTCCCCACCCTTAGTTTAGTTTGTGAAAAGGCCTCTGGGTAGGATTGGAGTGTTTCTGGATTATGACAATGGAGCTGTGACCGTCTCTGATGTTTTTGGAAGTTCCCTTCCTTCCATCTTCTCCTCGCCTCTGATACCATTCCTTTGCCTTAATCTCCATGAATTGTCCATTTCTCAGACCATTTATTGAAGCTTCTTCTCTGGGGATATTGCTGCCTATCACTTCATGTGAGACCACAGGATACCTGACTGTGTCCAAGCACATTGTTATTTAATGTGACAAAGTGATTGCATAGGAGAAGGCAGTGGTGACCCACTCCAGTGCTCTTGCCTGGAAAATCTCATGGACAGAGGAGCCTGGTGGGCTGCAGTCCATGGGGTCGCTAGGAGTCGGACACGACCAAGGGACTTCACTTTCTTGCATGGAGAATCCCAGGGACAGGGGAGCCTGCTGGGCTGCCATCTATGGGGTCGTACAGAGTCGGACACAACTGAAGCG

>LNC_001699

AGCTCCTCTTGGCCATCCTGTGCCCATGCAAGCGCCACTTCTTGGAGGTGGGGTAGCTCCTCCCGGCCACTGCCCCTGACCTCGGGCATGTGGTAGCTCCTCTCGGCCGCTGCCCCTGTCCTCAGACATGGAGTAGCTCCTCTTGGCCGCCGCCCATGACCTTGGACATGGGGTAGCTTCTATCGGCCACCGCCCCTGACCTCGGATGTGGGGTAGCTTGTCTCAGTGGCCTTTTCTGTGATTATGGTTTCAGAGTGTGTGCCATCTGATGCCCTCTCGCAACACCTACCGTTTTACTTGGGTTTCTCTTAACTTGAATGTGGGGTATCTCTTCACAGCTGCTCCAGCAAAGCACAGCCGCTGCTCCTTACCTTGGAGGAGGGTTATCTCCTCACAGCCGCCCTTCCTGACCTTGAACGTGGTGTAGCTCCTCTTGGCCATCCTGTGCCCATGCAAGCGCCACTTCTTGGAGGTGGGGTAGCTCCTCCCGGCCACTGCCCCTGACCTCGGGCATGTGGTAGCTCCTCTCGGCCGCTGCCCCTGTCCTCAGACATGGAGTAGCTCCTCTTGGCCGCCGCCCATGACCTTGGACATGGGGTAGCTTCTATCGGCCACCGCCCCTGACCTCGGATGTGGGGTAGCTTGTCTCAGTGGCCGCCCTTGACCTCGGACGTGGGGTAGCTCCTCTCAGTCGCTGCCCCGACCTTGGGCGAGGGGTAGCTCCTCTGGCTTAGACTCCACAATCCCAATGTAGGGGACACAGATTTCAATCCCTGGCCAGGGAACTAGAGCCTACATGCCACAATCAAAGATCCTGCATATGACAACTAAACCTCAGTTCAGTTCAGTCGCTCAGTCGTGTCCGACTCTGCGACTGCATGAATTGCATCACGGCAGGCTTCCCTGTCTACCACCAACTCCCGGAGTTCCCTCAAACTCACGTCCATCAAGTCAGTGATGCAAGCCTAGGTGCAGCCAAATGAATGAATAAGTGAATATTTTTTTTAAAAAAGAAAAGGATCACATTTCCCCCTTTTATGGTTGGAATTGGGAAATAGATGGAAACAGTATAGAGCACATTGCAAAACCTGAAGACATACTGAGTCATTGTTCTTATCTCCTTGTCTCTGAGTGCTGTAATTTCCCTATTCCTCACCGCCCTTTATTGTGGTGTGTGTGTGTATTTTCTTTTCCTCTCTCATCTTCAAAAAGGCCGGGATAGATTGGCAGCGAATTATGAATTCTGTTTTGGACACGTGGAGTTTACAGTGCAGGCAGAGTATTTCAACAGAGATAACGTCTGAGAAGATGGAGATAGGGAGACAAATTAGAACAAAGATTTAGGATAAATTCAGTGAGAAGAGAAGTTTGAGGCTATGAGGTTGCCAAGTAGAACGTGACAGAGGAAATCTGGAGAAACCAGTTTGACAGGGAAAGAGCTTCCTTTGTCTTCCTAGCTTCAAACTCAAAACACTTCAGTGGCAGTCATATCAGCATTTACTGGTTTCTATCATCTGTCATAGTTACATGTATCCAATTCTAGACTCCATCTAGATTCTTACATAAAGTAGAAACCATGTGTGAATCATCCTTATATTATACCATATGTCCTTATTGAATGCCCAAGACCTGGCCGAGGTTTGATAAATACTTTGCTGAATGAATTAATAAAATGGTGGAGATGTCATCCTCTTCAATAATTAATGAACCCCTACTAGGTGCCAAACACGGCATTAGGTACAAATATTTAATCTGCAAGATCTTAAATGTTGACTTTTTAGCCAGCCACTCATTTCTCATTTATTCATTAGCTGACATTTGATGAGTGCCTTGCAGGAGTCAGAAGTCAGAAGAAAGGAATCAAAAATGGTCCCCACATCAAGGATAAACTTGCTCCTTTCTTTCTCCCTCAGTCATACCTTCCACTGGAAGCAAAACCCACCAGTACCCTGGATTCTGACCCTACATGTATTAGTAATATGAATACCAATTAGGGGGTTGAATGACTCTGTGGCATTACTAATAACAGTGTGCTCTGAATACAAGTAAAAGAAAAATTCTTCAAAATTATATTCCCTGATAGCTCAGTTGGTAAAGAATCTGCTTGCAATGCAGGAGACCCTGGTTTGATTCCTGGGTCGGGAAGATCCGCTGAAGAAGGAATAGGCTACCCACCCCAGATTCTTGGGCTTCTCTGATGGCTCAGCTGGTAAAGAATTGCCTGCAATGAGGGAGACCGTGGTTTGATACCTGGGTTGGGAAGATCCCCTGGAGAAGGGAAAGGCTACCCAGTCCAGTATTCTGGCCTGGAGAATTCCATGGACTCTATAGTCCATGGGGTTGCAAAGAGTTGGACACAACTGAGCAACTTTCACTTCACACCAAGAAGTGACAAACCTAGGAGGATATTAGCTTCAGCTAGAGTGTAGTTCATCTCAGAGAAAATTTTAATAGACGATCTGAGCTTCTGAGTTAAATAGTTTTGAGTCCTGTCTGCCATTTGCAGATTGTATGACCTTGGTTGATGAAAGTACTGCCTACCTAATACAATTAAACAAGTTAGAAACACTCAATTAAATTATTACTCAGTTCAAAGTATTTTCTATTTTCAAGGAGATTGAGTAACAACTTTGTCAATACCTAGTACACAGCCCTTAAAATGCATTATCTAGTTTAATTTTTGCCCGATTGGCACCATTCCCAGAAGCCACCAGCCAGGCAGCTAGCTGGGTGGGGACATGATGCCACCTCCCAGCAGCCCATCTGCCTTAGAATCCCTTGAGCCTCCAGCTGCCCTGCAACTCAGCCCAGATCACCATGGGGCCCAGAACCTGGCCCCAACCACCAGCAAGCCAACACTAGCTCTGGGACCCAAACTCAACCACCAGTGAGTGGACACCATCAGCAGAAGCCCCTGGGCCACAGCCTCCTCTCTCCCACCCCCCAACCAAGGAGTGAGCTGACATCAGCCCTGGGACCATCGTGGCTCCAATGTACTCACCAGTGGGGCACCAGTCCCAGGACTTCCTGGGCCCTAGCCCTGCCCAACTGCAGCCAGCTCCAACCCCAGAACCCCAGGGCCCTGCTGCCAGAGACTGGGATCTGGCTCCACCCACCAGGGGGTCAGTGCTAACCCTGTGTCCTCTTGGGCCCCAGTGGGTGGACACTAGCTCTTGGCCCCCCCACCCAGCAGCCAAAGACCCCAGGACCCAGTTCTGGACCCTTGATCTCACCAACTAGTGGGTGGGTACCAGCCTCAGGACCATGTGGCTCTGGCCCCACCCACCAGATGCAGAACTAGCATGGCACTGCAGCAGTCTTGTTAATTAAGAATTAGACCCACCCAACAGCAAACCAGCAACAAAAGTGGGATTCCATCAGCTGTGCAGCCAGAGCCATGAGGATCTGGCCCTGCCCAGCAGAGACCAGCAGCCCAACCAGATCAGGGACCTGCTACACCTGCCAGCGTGCCTACAGTAGGCAGCCAGTCATAAGGGCAGAACCCATGCAGCCCACATATGCCGGGAGCCAGCACGAGGAGTCCCACCTGTGGCAAAGGTCACGAGGTTAAGGGGCCTGACAGGCAAAGGCGAGTCAGGCCTGAAGGGAGCCTCGCTGGATCTGCTCGTGCATCTGCCCCAAAACCAGAGTGTGCCTGCCTTACTGCATTGTGCCTTTCACCTACTCTTCTAACATTAACAGGGGGCTATCTCCCCACCACCTTTTTCTGGAAAAAGTTAGTTAATTTAGAGCTTTTAGATAATAAGTCTCCTGGGCATAATAAGAGTGTTTCAATCCAAAAACCCCTCTGATGGCTTTCTAGCCTGCTTTCATATCTTTTATTTGTTGATATAGTTGGCATGTAGAAAAAACAGGTAGTAGCCCTGGCATTAGCAACATTAGATCTCTGAGTTAAGTACTTTCTTTGTTATAACCCACTGCACCTTTGTGCTACAGGAATGTAACTTTATTTAGTACTTTGAGGGTGATGCAGATTAAAGAAAAAACACTTCAAGGGAAAAAGAGTTTTCTGGTTGATAGACATTTATCTAGGAAAAGAGCCATAAAAATGTTAACAGGCCCCCTGGCCAGAAGATGATGTAAAACCACATAAGACCTTTTGTATACGGGAAGGTATGCAAAAAGAAAGCCTGGTCTCAATAAGGGTCAGGACTGCTGCCCCCCATAATTCTGCATATTCCATTATTTCTTTATGTACAACTTGGGGTATATAAGCTGCTTTTGAAAATAAAGTTGTGGGTCTGGCACTGACACTTGGCTCCCCCATGTTGTCTTTTCTCGTTTTTCTGGCTGAATTCCCATCTGGAG

>LNC_006006

GAGAGGGCATGAGCTGGATTCCACGGGAGAAAGATGAGTCTGCAGGAGTGTGGAAGGTGGACGGGAGGACCTGTGTCCGTGTGTCGCCGGTCGCCCACTGGTGTCTCCCTGTGATCCCCAGGTGCTGACCGCCTTGCAGAAGGACAGCAGGGAAATGGAGAAAGGAAGCCCGTCTCTGCGGCACTGACGAGGCCGTGGACTCGCGATCAGCTGGGAAGCAGGCTCCACGTGGCACCTGTGTAGCTTCGGGCGGATGCCCTGGCAGCTCCGGAGGACAGGGGCTGTTTCCACGTTTGATCCCTAATGCTGGAGTTGGCCTTAAACACAACACAACAAACGAAAAAACACTTTTAAAGAATTAAACCAAGGCTAAGCCTTAAGCTCAGTGGAATGAGGACCATGGCCAACCACTGCGTATTCTGCATGGTTTAAATAGGGCAGCGGCCAGTTCCCTCCCCTCATTTCAGCAAAAGTTAATTAAATTGTAATGCTTTTATGTCAACTGCCGGAAAGTACATTACAATTTTTCGTTAGCCAAGGTGCATGAGAAATGTTATTTGGGAGACCTCAGAGATGGCATTGGCTCACATCTACATATTTTAGTCCGAAAGGGACT

>LNC_004068

GGAAATTCTCACTAACATTAACCCTCACTGACATTTGTTTGTTAAGTTTGCAAATTCTTTATATTTCCAGCTGTTGAGACAATATTTCTGAGAGCTGAGGTTACCTCTAGTGGCGAAACCAGAGCCAGCTATTAAGCAGCCAGAATGCTACAGTAATTGAACACATGACCCGTTCTTTTAGCCCGTTTTTTGTTCTCTTCCAGAAGTTGTAGACGTCTATTTAGCTTGATTATCTGTAGTCTTAATGAAGCTGCATCTGCAACTGTCATGTCTTCTGAAGTTCCTTCAATCACTGCATCTATATTTGAAATGCCATACCTGACGCTGTCAAGATGAGGATTAGAAGTGGCAGCAGCAGACCCACCACGCAACACAGGGAGCAGAAAATGTCAGCGAAGACATCAATGTCAGAGACGAACACCAGCATGGAGCTTACAGCTCAAATGTACACTGTGCTACATGTCGATTACAGCGCCATGAGAACTGGGGAAAGAACAGAACGGGAAAATGTTTGGGATAAAATATCAAGTTAACAAAGCAGTCATACGGCAGATTTTCATTGTGCTTGCACAAGAAGCCGAACCACAACTGAGGTGGTTTTCCACCACAGTGAGATCACCAAGGGACTCTCGGAAAGATCCAGCCTCTTCAACAAATCAATGACATGAAATATGCTGAGTATTAAGAGATCTGAAGAACCCTAAGGAGCTTCACACAGTGTCCCGTGACCACCTAGAGGGGTAGGATGAGCTGGGAGCTGAGACAGGGACATATGAATACCTATGGCTGATTCACGTTGATGTATGGCAAAAACCAATACAATATTGTGAAGCAATTATCCTTCAATTAAAAATAAATAAATAAATAAATAAAGAGCCCTAAGGAACAGAA

>LNC_004817

GGTAGCCATGGAGATCACATACCTACGGCTGAACAGGCCCCCCAGACTGGTGGACACCAGATTCCATACCAAGATTTCCCTTCGCCAGAAAGAATGTAATAATTCCCTATGTAGTCAATCACCTTTGTAATCTTTATAGCACCCATTGGTGTAGGTTACAATGAATAACCAGTTGACCCTTCTGATTATGAACCATGGCTGTAACCTGATTGTATCTCCCTTGAACATTTTCCAGGCTAGGTTTAAGGAATTTGGGGATGTGGGCTTGAGCATGTTCACTTAAAGTATATAAGGTTTTCACAAAAGTCAGTCAGTGTCCCTGGCTAAAGAGGAAACTCTGCCTAGGACCCACCGGTGTAATAAACTACATTCCACA

>LNC_002885

AAAGAAGGACACCAGAGAAAAGCAAGGAAGGCCCCCAGTTCCGTCACATGCCAAGGAAATAACGCCGTGTCGAGCAGCAGCCAGTGTCTCACAAGCTGCCCTTTGAGCCTTGGGTCCCAACAGGGACAGCTGTTATCTCCCGCACAGGTTTGACAGACAGACTGGAAGTTCCTCAATGGCAAAGCACGCGATTCTTCAGTAGTTTTTCCCAGCTCAGAATACAAAGTCTGGATGGACTTCTTGCCAGCTGCCAAAAGTACTTCGGGCTGTGGATCTAGCAGTTTGAACCATGTGAGTATAAGTACTAAATTCTTGCCCAGTTTGCTCAGAATAAAAAGAGGGCCTAGGGTAGCTAACGGGGACGGGGACGGGGACAGCGATTCGGTGAATGCTGTTTTCTCACGGCAGGGCCTCTGTGCTCCCCCAGCACTAAGAATTCCTGAACAAATATGCGAGCAGGAAGGGAACGGGTGGGTGAAAGTCAGCCCCATCTCAGAACACAAAAGTTGTCCTGTGTTTACGTTTTCAAAAAGCGAAAATCCAAAAAAGGATTGAGATTGGAAAATTCCTGGGGAAACAAAAAGCTTAACCTACATTCCATGGTTGTATCAACGTAAGCCTTTCTGAGCAAGGAGGTCAAATTTAACTCTGCTTCCCTGTTTACCAAACAAGTCAACTTATTTTGTATGTATAGCTATAAACTGTGTGCAAGTTTCCTAAGTCAGGCACTCAGGATGAATTATGAATGGAATTAGCCAGCTTGACTTGGAATTTCCTCACTTTTACTGGTCTTTTAAACAAGATTTTTATACATTCATATGTAATCAATGGGACCAACACATGAAATGATCCTTAACGTAAAACACTCTGTTTTAGAGTGATTTTGCAGTTAAGGAAACTTAAATATGAACTTCTAATGTGGAAAAGGTAGTTTTTACATGCTGCTTTAAAAATGATCTGGAAAACTGATGAGCTGGTCACAGTATTTCTGTCAGAAACTGAAATCAAGTTTATATTTTAAGGTACAATTTTAAATACTTGCTTTCTTTCATGAAGCCAGTCTGCTGCTTATATCCTGAGCCTTAGTCACCTTAAAATACATAAATGTGTTATTTGGCTATTGAGTTTTTAAGTTGCAGGGGAGAATAATTTATATATTCATAAATACAGAGTATAAGGAAATCTTTCCTGAGTGCAAAACTACCTTTTAAACTCTCACTCATCTAAACGAGTGAATGATACAAATATACCTCTTGATTTAATTTTGGCTAGTAGTTATTTTATATCTTAATATATATTCTTTCATATTAAACTTGCACCAGCTTAAAATTTCCCTATAAACCGGCATGTCCAAATTGATTTCCATAACACTTTGTGTACATAAAAACTAAGAGATCAGGATCAATGTCAAGTTTTGGAGAGAAAAATGAACTTGGGATTGTTTTTTACTTCAACAAGGCACATGTTCAAATTGATCCCATATACATATAAAAGAAGATTAAAATATTTCTCAACTATTCATGCAGGAAGTAAAATGCAACATATGCCAACTATATTTTTATTATATAAATAATATAACAAAATACTTGGAACGGTTGAAAGGCAATCATTCAAGAATACATAAAAATGTAAAAGAATACAAAGCTGTTGGAAAAAAATACTTCTTAGTCGCTTAGAATCTAATTACTTGCACGTTTTAATGGTCTTGTATATTTTGGTTGTAAAATGTTTCTACAAACTTCTTGCCTTTTCAAGTACCTCAAATACATGAAGGACCAAAAATATTTTTTCCTTCATAATACATTCAAAAGTATCCATCATCTTGACAGAACTGTTATTATAGTAATAATTTTCTTATACCTGATTATAATATTTCTATAGAATGGAAAACTCTGACTTTTCTTCGGAAGGGGGAATAGTTAGGGTTTAAAACATGTTTTAATAGTCATATAAAATGTATTCATTCCCAAGTTTCTTTACAAAAACCTTTGGTATTACAATTTTATCTAGATTACATTTATAGTTTTGGAATAAACCAAACCTCTTGAATAGGGTTGTACTAATCTTGGTTAAATTAAAAAAAAAATTACTTCAGTGATATCTACTTGAAGAGAGTTAAAGAAATATTAATCCTGATGTTTTTAGTAGTTATGGAGATACTTTTTTTCTGAGGGACAGCTGAACTTCAATCTGAAATGTGAACACAGAACAAAATAACACAAGTATAAAGTTTCCTTCTGAACTTAATAAATCAACAAAGAAATCTTTATCGTGAAATTTTAAATACAAAACTTTTAGTCTGAACTCTTGAGAAAATCCTATGCAGTAAAATATTTGTTTCTATATTTGCTCCTTAAACTTTCTTGTCCTTCATTTTTCAAAGGAGCACTGACTGACCACATTCACAACTGCTGTCTGCAGGGCATGGCTCAAAGTCACCGCCATGTCATCTGTCGCTTCTCCCCAGCAGCAGCCACGGTTGTCAAAAAGAAATTTTATTATTTATATCCATTCTTTTTAATTCTGTTTTTATTAGCTCTCAAAATCTTTGACAGCAAATGTCAAACCACATTTAAACTGTTTTGATTTTTAACTCCATGCTCCAGGCCAGATTTCAAATACTTTGCACATGATTAAAAAAAAAAAAGGGGGGGGGTTGTTTAAAAAACTGTTAAAGAACATTTCATTCAGATAACTTAAGACCAAAAAGTCTAAAAAATGGACTATAATCCCCATTGTAAGTTAATTATAGTTAGTTTAAGCTAGAAGTGTCAAGTGAATACTCCTTAACGAAAGATCCTCGGGTCCTCCATTTGCCTTGGACATTACCTGGCCACACAAAAAATCAAATGCTCTTGCCATCTGGAAAATTAAAAATACAAGGTATTCTCTTCCTCGTAACCCGCACAAGCCTCGACAGGAAACTAAAGGATCTTAGGCGGTAACTTTAACTTACCTCTTCTGGGCTTTAGCAAGTGGTTTACTTAAAAGTATTTACTTCTTTGCAAAAGGCAACTCATTCTTAAATGCAGCTGAAAACCTTGAACAACCCTGAGAAAAAATGATGCCCCGAAATGTGTCACTGCCCATTTACTTCCTGTTTCGCCAACTTATAAGTAATTCACTCCTGATACTGACTCCTCAGATGGCAGCGTTTGCATCTTTTTGGACTACAAAACGTGGCCCGTGGTTACTTCTCGCTTACAGGCACCCATCTTTCATTGGGCGCCGTCACCGTCATCTTTCTTCCTGGAAGACAAATCCTTTGCAGATCTCGCACCCCTCTTTACAAACCTGCGCGGGGCTTTCGGGGGGAGAGGGAGGCAAGACTTTGGTGGGAGCTGCTTCTCTTCACCAGAGCGAGGGTTGACTGCCAAGGCTCGGGCTGGGTTCTCCAGCAATTTCCTGCTGATTCCAGATTCCACTCGAAGCAGCGTGGAGAGATGTCCGACTTCTTTTCCGTGCGGAGTCTCCACGTTGGACTTGGAGAGCCCGTGGCCGTGAGCCCGGGGCTGAGTCCCTGTCCCAGGCCGACGGCCAGCCCGAGGGGGAGGGCCGAGGAGAGGGGTCCCCGGGGCGGCGCGCCGCGCTGGGCTCCGGAGAGGGGGCTGCACGAGGGAGAGCTCGCTCCCACCGCCGCGGGCTCCAACCCCGGGGATGCACTTGAACCCGCCGCGAGGCCGCCCACTTGCCCCGCTCCGAGCGCACCCGCGCCGGGCGGAGCGGGACGCGGGCCTCACGCGCTGCTCTGCCTCGGTCGACCGAGAGGCCCAGCCCGGCCCCCGGCGCCGGGGGCCCCACCCAGTTCCCCG

>LNC_004624

CCACCTTCCATAACATCCCACAGAGTCAGATACGACTAAAGCAGCTTAGTACTCATGCACATGGTCATTTATATCATTTGGGCTGGGACATGATGCATCATCTCAGCATACAAGAGCATCTCGAAAGGTGCCGGCAATGCACCAATATCTCTCTGGCAGTCACATCATATCTCTAGACAGTCGAAGGCCAGTCCCAGGAACATGCCAGAGGAACTGGATCTGATGGTGTGTGATCCAGAGCCCTCTCTACATTGAGGGAAAACAGAAGTGACTATGCAGGGCCTGGAAGAGCCTTTCTTGGAAAAGCTGAGTAAGCAGGTCACACATAAATAGGCCCTGAGGACAGAGCCTTCCTGCCTCTCCCTCTTCACATCACCATCATGGCTGAGACTGCGCCAGTCTTGGTTTTGTGTCTGCCCTTACTACGGGCTTGTTTCCATTCCTTTCCATTCTTGCCTTCTGAAGTACCTGCTGCTTGTACCTCCCCAGTGATTTCTTTCAAGTAACCTTATGGGCGACTCTGCCTCTCTGATATGAGTCCTGAGTCTTCCCTCTGCCCCTTCTGTTCCCAGCCCGTGAGCTTACAGTGTTGGCACAGCAGAGGCAGCGGCAGATGCTTGGAAAGTAGAGACAGGGAGAGAGAAAGAGAGTTCAAAGAAATACTAAGACGCTACTTCACTTGAATACCTACTTTTTGAAAAAATTAAGAAACCATGAAAAACCTTTAGGGAATTTATTTTATTAACAGTGAATTCTAATCTGTTTCGTGTCCTGCTCTTGTTTAGTCCAAGTGCACAAAACATGCCTAGAAGTCAGTAGAAAAAGACCAGAGCGCGTGTGCGTTAGGTTAGTCCCTTTAACCAGAAGCTTCGAGAGTTTCCAGAGGTGGGAGCGGATCCTTGCCGCCTGCCTGGCTTCATTCATAGGTGCTTTGTGTCCCCTGCTGCCCCCATCTCAGCAGGGAAACCACATGTCATAAGACAAGGCAATCCTGGCTTCAGAGACTAGCAGCACTGAAATGGAAATTATTGATGGTTGAAGGGGTCAGATTATGGCGCAAATCTGAGCAGTATTTGGGAACATAAACTACTGTGAGTTTAATAACACCAAACAAGAACAAAATGTATTTTTAAAAGTATTTCATCCATGTCCAATTTTTCTGGCCATTTTCCTGTATTCATTTTCTGTGAGGGGGATACTGAAGTGAAAATAGAAACTATTATTACCCCTATATTAAGGCACTTACCTTGATCATGAAGAACATGAGAAGTCATATCGGATAGTAAACATCAAGGTATGAAATAGTGTGTTATGGATTGCTAGTTTTGAACTTCAGTGCTTCACCCACTAAAGCCTAAGTATGTTGTCAAGAAATAAAGTGAAATGCTTGGTTCATTCTAAGTACTTAGGACTTGCAGCTACTGTTGTCACATTGAGGATACTCAGGACATTGAGGATACTCAGGACATTCTTTTAGTTGTGTTTCCAATAGTTCAGGATGGGTTTGTTTTGGTCTGTCATATTTTTTTGGTGATAATTTGTATGGCAAAGAAAACAGTGAGGTTGATAGCCTTTACTGAGTATCTCCTATGTGCTAAGCATTGGGTGAAGAGTGTAAGGGGGGACGTACCATCAGCATTCCAGTTTTATAGAACGTGCTTGATAACAGAAGGTTCTTGTCAGTTCCCAGCAGAGACCATAGTCTCATCAGAGCCGAATGTTGGCCTTAAGTAATGTTGCAACAATATTTCAAGGCTTAAAAAGCTTCTTGTTTTACATTGTTTATTAGCTGTGATAGATAGCCACAAAAGGGAAATGTTCCTTATTAAAAGAGGGGGTTTTCACAGAGTAAAAAAGTAACTCAGATTTTCCTAAGCTTGAAGAATTACTTTCATATTTGATGTTAAAGATTTGTAAGTAAATATTTGTAGTTTGTAATCTACTGTTCAGTCCTCAAGGACAAAACAGTATAATATAAAAAAATTAAAAGTAATATTCCCTTTTAAAATGTATATAATCAACATGTTTCATGTATTTCCTGAATATTTGTTATTTTCAGTTGTACATGATGTAACTTGCTATTTCAGTAGGAAATGAACCGCTTAGGCAGACACATTTTGAAGGATTTCAGTATTGGGGGTTTGTCACCATCAATAACTGAAAGAAAATAAATCCAGTATCTCTGATTGAAGAGGCAAGTAAGTATATATGTATAATCAGTTTTCAGCTTACCACTTTAATAGAAGGGAACATTAGCATGGGTAATCAAAAGTTTCCAGCAACCAACACAGCCAACTTTTTTTTATTATATCAAGGAAATAAAGGCTTAACTTCTCCTTTTTAAAAAATTTTTTTTTTCTTTTCACAGTTACTTATCTTCTGTTTAGTTTTGCTTTGGACTCTTTGGTTTCTGGAGTGCACAGAGATGGCAGTGGATTTGGAAGTGGCTCAGTATATATTTGTCAAATAGAGGAACATCCTTATAATCTAAAATATGTTGTAAATATTGTAAAATGTTTGAATTAATAGTCAATTATGTTATTGATTTTATTACATTTCCACTGTAGATTTGAATCCATTTGCCTATTCTTAAAACTATAAATGTCCAGGGTAAATCTTAACACTTCAATCTATTTTAACAAGCCCTCCTTTCTCCTATCCCCCACTATTCCTATCTTTTTAAAGGTGAAATTAGCATAGAATGACAATCAAAGATACTTGAAGGGGAAAATAAGTCACAGAATAATTTAAATTTGACTGTCTGAATTTGGAATTGGAAAGAAAGTTAAAGGACATTTACCTTAACCTTTTCAATCTACAGACGAGGAAACTAAGTTTCTGAGAGCTGATTGGATTGCACCATGCCACAGAGTTGTTACAGGGCAGAACTAATATAGATAGACAACGTTTCTTCAACTGAAATTTTTAATGATTTTTTTTCAGCTTTATTAAGATCTAATTGACAAATGATATTATCTTTAAAACATAACATCATCATGATTGATGAATATGTACCTTGGGAAAGGACTCCCCCCACTTGAATAATTTTCAAAAATTAATTAATACTAAGCCCTCAGGCTTTTTTTGTCTTTTGGTTTTTGTCTTTTAGCACACTACTTGGAAAGTGTGCGTCCTTTAGCAAAAGGTAGTGGAAAGCAAAACCCACTCATACCAGATGCACTGTTTTTTCCATTCTTCTCGACTGGGGGCCGCCTGATGGGTACAGATAGGAAAGAAAGAGAGAGACCCAGTCCTCACCTCAGTGTCCATGAACGTTTTATGGGATCATTTTTAAACCTGGAACCACTTGCTTTTCTAACAGTAGTTGCTCCCTGAGGAGTTCCATGTTTTTCTCAGCTGCTCGTGGCCCTGTCCAGCCCAGAAAACAGCACCCCTAATTCCTATATTATAGGGAGGCCTCTGGCAGAGCCCTCTCCTGTTTAGAATATTATCATCCACATAATTATTTTACTGGTAGGGAAGTAGTTTTAGTCTTATCCATTAGCTTAGAGTATTAGTGTGCTGGGCTTGCTGAGGTTACAGCTTTACGTTGGACTTCCTTCCCCCTAGCCCATAAATCATTTGGTTTCCCAATTTATTAAAAAATTGAATTCTTAGAACCAAGCACTATTCTCGTGCAGATGGCACCAAATGAATGCTCCCGGTTCCAAGTACAGTTAATGAGACAGGGTAAACAGGGCAGGAAAATGCGCATCATCACCAAAAAAAAACCCCATGTGTAATCCCACTAGTCAGAAGGTCATGTTTGTCAAGTGCAGACCTTACCACTCTTTTATCCAAAAATGTATCTTTCTTAGAAGCTGATTGACTAGATTAGTATATACTTGAGTCATACGTTAAAAAAAAAAAAAAAAAAGCTACAGAAAATATACCCTGTGACCCATGTGAAAAGTATTGATTTTAGACACAATACCTGGTAGAAGTAAATGGTTTAATCTGACCTGGAAAAGAAGTAACCTAAATACACCTAAAAATAACCTCTAACAATTGAGATACAGTGCTAAATAGTAAAATCATGTTTCTGCCTTCTAGTATTGTCTTGACACCAGCATATACAGTTATTATTCAGTTAGAAATATTATTTTGACAGCATTCTACCTGATACATATTTTTTCAAAAGGGTGGTAAGCCATTGGTTTACTTCAAAAAGAAATGTATTTGTTTGGCTGTGATGGGGCTTAGTTGCAGCATGTGGGACCTTTAGCTGTGGCGTGTGGGATCTAGTTCCCTGACCAGGGGTCAAACCTGGGCCCCTGCATCGGGAGCACGGAGTCTTCGTCACTGGACCGCCAGGGAAGTCCTCCGTGCTTTTTGACAGATTTTTGACAGACTCCTTAGGGAACGTATATATAGCAAACTGTGAAATGTGACAAATTGCAACAAAATGATTGTCACATTACACCTGAGCTCCAGAGTTTGTCTTAGTTATCTGAGTATTTCATTGCGTTTTGAGAAAATCTTGAATTATTCTAGTAACTTAATCCTCCACATCACAATGTATAGTGCTTCATTATGCATTCATTTTTAAATAATTTATTTTAAGTTTGAAAGATAATTATGTTTGTTTTGCCACAAGATGGTCTCATGAGAAAAGTTCCAGTTCTTGGGGCCAGGTGTAGTTGCCAGACAAAGACTTTAATGTCATTTGGCTTTTGTTTCAGAATTCGTTTGCACTGGTTATTTCAACTTAGATTAAATAGGAGGAGGAATAGTACTTCCTTTTCCTTCCAATATGTGGAAACATACAGTATTTGTGTGTGCATGTGCTCAGTTGTGTCCAGCTCTTTGTGACCCCATGGACTGTGGCCCGCCAGGCTTCTCTGTCCATGGGATTCTCCAGGCAGGAATACTTGAGTGGATTGCTGTGCCCTCCTCCAGGGGATCTTCCTGACCCAGGGATTGAACCTGAGTCCCTCGTGTCTCCTGCGTCGGCAGGCAGGTTCTTTACTGTGGTGCCACTGGCACTCCTCTGGTACTCAGCATTTAAACAGTCCTGTTTTGAGTTCTTAGGTAGCCAGGAAAGGTAACCATGCTACCAGGAACAGAGTTGATTCTAACTTATCACTGTGTAGATCCTATCTTTAGGGCTTACTATATAACCAGAGGACAAGGGTGGGTAGATGGAAGAAAGAGGAATCCTGAGTAGTGGGTTGGCCCATAACATCTAATAGACAAGCCAGATGAATTTGGGCCAACCTGTAATTGTTTCATATTTCTCTGAAACATGATGGTTTGCTTGCCACAGTGCACATTATACCTGGAATTTCATTGCATCTTATTTGAAACCAGTGAGGGGAGTCACACAAGTGGGTGCAAAGAAAAAGGTACCCCCCCACAAGCTCTGTATCACTCAGACTGGCTTTGCTCACCACAGCTACAGGACATGGAGGGTGTCAGAAATGACTATTAGAAAAGTTTTAAGTGCCAGCACCTGAGAATGAATTATTTTTTAACTTCTAAATAAATGAGAGTAAGATCATCACAGAAAAGTAGGGACCTACTCAGTCACATTCTTACATGTTTTCTCAGGAGGGAAGAAGGGTTGAGTTTGAACTAATTTGCTGGGAATTGTGTTTAGAATGCATCCAGGGTATCAAAGTGTAAAAGGGGTATGCTGCCTCTGGCCTGGATTGTGGTGTGTACTTTACTAGCCAGAGAAATGAGGACAGTCAGCAAAGCCTGGGCAGGAGTGTTCATGGTGTAAGTCTGCCTCTGGGCTGCACTTTCCCCAGGGCCACAGTCTCAGTTACGCATGATTGTGCTGTTAACTGTTAAGGATACTATGCATTAAAAATGAGGACTCTGGATCCAGACTTCTTAGGTTCAGATACTGACTTTGCCGCTTAGGCAACTGACTTAATTTCTCAGATTCTTCATTTGTAACACAGGGTTGATACTACTATTTAACTTCCTGGGTTTATTAAGAGGATCAGATGAGTATAATGGGCTTCGTCGGTGGCTCAGATGGTTAAGAATCCACTTGCCAGTGCAGGAGGCCCAGGTTTGATCCCTGGGTTGGGAAAATCTCTGGAGAAGGAAATGGCAACCATCTCCAGTATTCTTGCCTGGAAAATCCCATGGACAGAGGAGCCTGGCTGAGCTACCAGCCATGGGGTTGCAAATAGTCGGACATGACTGAGCAACTAACAGTTTTGAAGTGCTTAGAACACTATCAGGCACACAATAAACAATGAGTATGTATTAGCTGTTATTATGTGACCCTATTTAATTTTTAAAATAGTATGATCCTCCATAACCCTTATGTAGGGAAAACAGACACTTAACTGGATGAATGAGTAAATAATAACCTGCATGGTATCTTTATAATTATTGATTGGTTATGTAAAAACATTTTGTTTTGTGAGTTAGTTTCCAAGATAAAGCTATGGAGGCACCACATTAATTAAATTGCATCCATCCATGATCCTCCTACCTCATCTCAAACATGATCCTTCATCTTCTTTCTAATAAACCATTTTTTTCCCTGGAACTCACAGAAAGGATTATGTTTTGCATCAGGAAAAAATACACACAACACACATAAGTGAAACAGTACTTCATGAACTAATAGTTTTACTAAGTGTAGTGAGCTCTTGTTTTCTATTTTATTCTCTTGTTTCATTTTTGTAAAAGTGTTGCTTGCAGTGCATTGAAATTATTTCACAGCCCACTAATGGATTACCCAACAATTTGAAGAACACTGCCTTAGACCAATTTGACTCTTGTCCAGAACCAAAAGAGGGGATACCTTTGATTATAACCAATATTCCCTTCCTTTGCATTTTCTTTCCTAACTTCCCCTGTTAGTGGAGCTGAGATTCACCAGCTTTAAACACACACACACACACACACACACACACACACACACACACACTTAAAAGACCACCAACCTGTTTAGTAAGGACAGAGTTTGCATTGGTTGTCATGTAAAAGGACTTTAAGCTTTACTGCATTTCAGATTCACTTTTAGCACATTTGTAATATTAAAATGCACAGAGCTCATATAAGATAGGACCCAATAATGATTTTCAGGAGAGAGTTTAAGGTTAGGTATGATAATAGTAACAGATTTCTCAGATTGTGATACCTAACTTCCACTCTGCCCCAGTCCTCCCTAATTAAATAAGGCATTACGAATGTCATGCAGAAAAGTGACTTTATCAATAGAGCAGAGCTCTAGAGAGCTGTTGGCATCCACAGCTGTCCATAGGCTCCAGTGTGTCCCTCTGGACCCAGCACCAGTTCCTGGGCACTGACTGCTTAGAGCTGTTTGTGTGCTGTGACCATAAAGCTGAACACACAGCACTACCCACCCCCTGCTGGACCCCGATTCTATTCTTACCAGCTGAGCCACAGGCCAGACCAAATAACCTACTTCTTTGAAAGACAGACAACCAAATTCTGACCACGTTTACCACACAAGGCCTTACCCATGACCAGTTGCATGTTGCCTCTGGTCAGCCCCCAACACCCTGCTCCACACAGCAGCCAAGCTGTCCTGACCCCAGTGCTGTGTCTAATCCAACACCTTTTCCATCAGCCTTGGTGGAAGTCTGAATATGACATTCTCTTCCTGCCCTGCCCACCCACCTTTGGCCCTTAGTCTTTGTTAAATCCATATGTATTCCTTTATCACATTTCAAGTAAACCTCTTCTAAGACTTTTTTCTTTCTTCCCTCTGATTTTGGAGAGCCCCCAGAGGTGCTGGTCACAGCTGATAAGTCAGGAGTAGGGATATGATCATTATCAAGAGGAAAGAACGTGGTATATTTGAAGATAGTGTTAAACAGAATAGTCAGTGTCATCCACTTTTCAGATCTCTTAAGTGTCTTAGTCAATTTCATCCTTTTCTGATTATACCTTCCTTCACATTTGCATAGAGAAAGAAGAAGCATTCCTGGTTTGTTTTATTCCTTATATAATTGTTGGTTTTGTAACACAGTTCCAGTATACATCTCATTTTCAGATATGTGTGTGTGTGTTAGTTGCTCAGTCATCTCCACCTCTTTGCGACCCCATGGACTGTAGCCCACCAGGGCTTTGGGGCCTTTCCCCTATTCAATCTGGTTGGTCCCACATGACTCAGGAGAGACCATTTCACTCTGACCACTTTCTCACACGTACACTTGAAGTGTGATGGCTTTGGCTAGTACAACATACTGTCTTATTTTAAGACTGGGAAATGGCAGTCAGAAAATGTTTAAAGAGAAAAGGGCTAGTACATTTTTCTGATGGTAGAATTTTAGTCGTATTGGATTTTCGTGACGTCGTTTCAAACTGTGTCCTCTGATGTCCTGCTATTAACATTGTATTTTTTAAAATAATGGCTACCTAGCAACTCTTTAAATTCATTCCTCATTTTTAATCGCAGGGTAATAACTTTTCTTTTGCTAAAGTTTTAGTATATAGTCTGCAAGCTACCATACCACAGGTAGCAAGCTAAAGGCCACTGTGAGAGTGAAGTAATAATACATCATGGCTGTGGTCTCACTCATTGAAAATTCATTATCCTGACACAGTTTTGTGTTTAAAATGACCTTGAACTTGCAAATATGCTTATTGTTGCCAGTAGAGAAACCAATAGAGATTATAATTTTTCCACTAGGGTCCGCAAAAACCAAATATGCAACAGACTTCCTAAACTGCATGTAGTCCGCAGCATTTGAAGCAGAGCTTGACATTGTTTTTAACAATTCTTATATATACCCCCCTTTGAGAGAGAAAGGCATGCATAGAATAAAATGATAAAGTATACATTTAAAAAATCATCTTAGATCTGAGAAAAGATAAAACAGCATCCTTGGGGTTTGGCAGTGTTTCCTCAGTAGCCATCTGGAGGGGTGAGATGAGTCAGCCGGCCCAGATCGGGAGGGCTTGCATCCTATAGCCCACCACATCAGAGGATGCTCTGCTTTCATCTGGTGCCTGTAGTGGGCTTTCACCTAAGATTTCATTGAGAGGTGGGCTTCCAAAAGCAACCTGCAAGTTTGAGGACTCCTGCCCCGATAGTTGCAAATTTTACCTGTTAGGCGAAATAGTTGGAATACAACACCAAATACACTAGATCAGCTCATAGTAAAAATTAAAGCTCTAAAGTCAGCTTCCTTACAGCCCTGTCTGTGTTACAGTGTCAGGGGGGTGTAGTCATCCTGAGACTCAGTGGGGGAGCATTTGGAAAAGCTTGTTCATAGACATACTGTTTGCTCAGTTGAGCTCCTCTCATCCAGAAAGCTTCCTTAGAAACTCTTTGCATGTTGGAGGAACAGGTAAAGCCTTTTCTATTTAGTAAAACATAGTTTTCATCCTGGTTGGTGTTGTTCAAATAACATTTTTCTGTCCATTATAAAATGATGTCCATTTATAATGATGGTGCCTCTCCCCTCTGCCCAGCACTTTGCACTGCAGTGAATTGACAGGTTAAAGTGGAGGACAGGTGATGGGAAAGGACTCTTGAGTTTTCCGAAAGTTCACACAATCTTTCACTGGATAAAGAAAGAACCAAGAATGAGAAATACAGAAAGATGATATCATTTTAAATGCATCTGACAGTGTTTTCTTCTTTTCAAAAAATCTTGTATGTTTAGCAGATAATGAAGGTGGAGGTGGAGTCTGCCACAGAGAATCCACTGCTGGCCAATGCCTACCATTAGACCACCGTTAGACATGGACTCCAAGTCTGTGTGTTCAAACACTTGTGATGAAGGGCTCCTTTTGTTTGTTCATTTTTTTCTTTGGTGAGACCCCGTGGCACAGAAAACTGTCTACATAGTTCTTGGTGGGGAGCCTGGAACATCACGAGCAGTCAGTTTTTAGCAGTGTGTTTTTCTGTTTTCCAGTGGTAGCATCTTCTAATGTAATGTTCACATTTGGCATCAAGTACTGAGTTCCTCAGCTTCTGCAGCTCAAGCTTTTGTCTCTGAGAGAGAGGTGGCAGGGAGGAAAGGCCAGGTGAACCCAGGCAGGCTCTCCTCTCCCTTGGAATTAAAGCCTCAGGAGTGAGGCAGCCTCAGTGTGACTGTTGCCCCTGATTTCAGCAGGAGTCAGAGATAAAATATCTGAATGTTAAGGAAGATAGCAGCAGCTGTAATCAACTGTTCCACTGACAGGGAAAAAAAAACTGATCTTCTAACTTTAAAGAATTTAAAAATAACCTGGAATGCTTGTTATCATGCAGATCGCTGGGCTGCACTCATAGACTGAGTCAGCCTTAGGTTATCTTGATACACATGGTCCAAGAGCTGAGTGTTGAAAGCCCCTAGTTCTAAGAAGATCAGTTACACTATTGCTCCATTATTCTTGGTTTTAATTTCTTGGTTGATCATCAGTGTAGCATGAAGTCTAAATTTCTTTTGAGAATTTTTACCACTCCTCCTTGGTATTTCTGAGCCCCGAAACCATGGCTTGGAATTCTTGGCTTAAAAACATGTGTACTGTGCCTGTACTTCTGTATAACTCTCAGGCAGCCACATAACACAAAAACTTTTGGCAGTGGCGTGAGATTCACTTGTAAATTGCAGCGTCGACAAAGTTCCTTCTTCATTGACTTTGCCCGATGAGGGAGTCCTCTCTCACTGTGGTTTGTTCTCCTCGTGGTTTCCGTAGCGTTAGGGAGAATGCTAGAGAAAAAAAACGAAAAAACGAACCCCGTCTAGTTTTTACTCACTTGGACGTGGTGTGTCTTTTGTCTTTCTGAGATGGGATGCGGGAGGCATGCCCTGAAGGTGGCAAATCAGACTTCCACACGCGTTACCTTCGCTTAGCCAATAGCTTACTCAGAACGTGTATATTATGATGTGCAGTTTCATTCATCAAGGGGATTTCGGGCTCCAGCAGCAAGCAGCCTTGAGTCGTGGGTGACTTCTGGGCTTTGTTCTTCGCATCGACACCTCCGTAGTAGCAAGAAAGTGCATCTGGCTGTTAGAAGAATTAATGGGAATCCAGGTAAGGGTTAAATGGAGAAATCATTCCTTTTTTTTTTTTCCTTTGGGTGGATTTGAGATTTTAGAAAAAATTTTTTAGTCCTTTGTTTGGGAAGGGCTCAGAACTATTTGTCTGAAGTGTTTCCCTGAGTTGCTTGATAGTATTACCTTGTATATCAACATCCCTTTGATGTGAAATCTACCCTCTTCAAATTGTAGTTTCATTTTCAAATATTAAAGCATAGGATTCCATTTTTCTTCCTGTGCATGAAATTTTTAAAGTAACTTTAGTTGCTAAGATATAAATTACACATAGTTCCCTAGTTCAGAACTGAAGGCTTTTTTTTTTTAGCAAGTGTGCTTTAAAAGTTGGGCAGGGAAAATCGGTGTTTGCATATGATTTGTGAAACAGTGATGTTAAAAATATATCAGACTTTCCCTTATGTCAGAGGTCAAATTTTGAAGCTTGCCGCTTAATCTGCTGAGGAAGGGGCCCTGAGACCTCTCCCCCTGCCGTTCCCCATGCTTTCTCCTGGTGGCACGAGCTCCTCTTCTCTCCCCAGGCGGGGACTCAGTCTGTGTACATTCAGTGCGGTGGCATTTTTCTTGAAGAAGTGACTAAGACTTGGATGAATTCTCTCTTTGAAAGAGGTGGTGGAATTGCATCAGAAAGGCATTTACAGTAAATGGCTACCCCCTCCTTCCTGTAATGACCACATATGAAAGAATGCATTAATTCTTGTTTTGAGGAAATGATAGTTGTAGAGTTCTATGGAAAACTCCCTTCTCATCACTAATTAACTAGATTTGAATGCTAAAAACCCCAGCGTCAGTATCACTTTGTGCTATCTCAGTGTCACAGAGATACAGTTTTTCATTCCACCCTCTATGTGGAGTAACCAATTATCTTGAATTCAGAAAGAGGAAATAATGTTGAATATGGCTACAGTTAATGGCACAAGCTTAAACTAATTTCATTTCTCCTAAGTGTGAAGTGTGTTGCTGGTTTTGTTCTTCAGGATTTAATTCTCTGTGGTAGAATATGAAGGCTAGAAGGAAACTCTCATAGGTCATCAGCCAGGGGTTCTTGTGGGAACAGTGCTGCCCCCTAGAGGACATAGTAGGAATTTGGTACCGTGTTTGGTTCTACAATGAAGGGCATTGAATGGGTGGGGATCAGAGATTTTATATGTTCCACAGTGGAAAAGACAGAAAGAATTACCTTGTATCCTACATCAGTTTCAGTTCCCCTGTTGGATAATTCATGCAGTGAAAGTTAGTTTGTAATTGTCCAGACTTGGACCTAAATCCATTTTATATAAAAATATAAGAAAACTTTTGCATACTTTTAATATACACAGGATATACTCATTTTCATTCTGGTATCCACCTCTAGCTCTCCTAGTTTGTTTTCCTGTACATAAAGAAAGCTTACGATTTGGCTTCTCTTAATCCAGGGGTTCTTTATAAGTAAGTGATAATCATCTATTTCATTATATAGTTAATGGGACCTGAAAATTATTTACTTACTGAAATACGTGGTGTTTTTATTATAAATTGCTTTTCTTTTAGGTTCTCCTTCACATTGCATTCAGGGTATTATATTGATGTTTTTAAAATATATGTATACTAGGTAATGTTTATCAATGAGTTGTATTTCAGAATAGTGAATGTAAATAGAGCAGAATATTTTGGTTATGAAAAGAAAGCTTTTGGACTAGTGGGGTTTTGTACCAGCGATCAGTCCCTCTCTCTGTTCCAGGAGGAACAGTATGCACATCACCCAGATGGAAGCGATGCTTGTCTTCTGTGTGAAGACCTCCAGGGATGACGTCACATGTTCTACATTAATGTGTTTTCATATTTTGAAGTCTTCACATTAAGCTAGTTCTTACTAATACTTTCCTGGAGTTTTTATTATGTCACTCTAAAAACACCATCTTTGGTTTCTCCCTTTCTGTAGGAAAGGACAGATAAATGTGGAGAGAAGCTCATAGGTAATCTTGCAAACTCACACAGGAGTTAAAAACAGACTCAGATTTTCTTGAAAAATGAGGCTTTGTGTCACCGTCTTCTTTCCAGCAACATGTTTTATATGTGCCGTTTTAAACTTCTGGCTGTAAATTTGCAGCATATCTTATGTAAGAAGGCACTATCTGTGTCACAGGATTAAGTTGTCGGAGTCACAGCTGTGAGGTCAGGGTACCCAGGCACCTGCATGCAGTGCTCACTGATACTTGTTTTGGGTCATGGTTTTAATTCTGTAGACCTTTTTTTTTGGATTGCTATTTCTTGTGCATTTTTGGGTGTGTTTTTTCCTTACATGGATATGATTATGTACAGGCTCAGTCATGTGTAACTCTGTGGCCCCACGAACTGTAGCCCACCAGGCTCCTTTGTCCATGGGACTCTCCAGGCAGGAATGCTGGGGTGGGCTGCCATTTCCTCCTCCAAAGGATCTTCCCAACCAGGGATTGAACCTGTGTCTAGGGATTGAACCTGTGTTCCTGCATTGGCAGGTAGATTCTTTAACGCTGAGCCACCAGGGAAGCCCATGGGTATGTTTATTGCTTCTCAAAATTTTTTAGGGACACTAATAAAAGCTGGGTTCTTGGTGAGATATCTTTACTGCTTTGAATTTTCTGTCTCTTCCAGTGTCTTTTTGTTGTTTGTCTTGGTTTTCCACTGTCCTATTGTAGGCTTTCTGTAAGTGTGTATTTATCCCTAACTCTTCACTCATGTACAGATGAGATGGCCTTCGGCTGCCAGGGCTCGCTCTCTTGAGTGCATCTTGCTTCCCTCTGGTGGCAGCCAGCCCTTGTGTGGAGAGTTGCTAAGCATCAGAGTGAAGAGGACTTGACTTGGGGGTTTTCAGAATCACATTGGCTGTTTAGTTCCCACTAGACATTGTGTTTCATTTTGTTAGAGAATCATGGCCCTTTTTTAAGTTTTAGTTTTGGCCTGGGGGTACCTATAAGGTCTGATGTTTCTTATAAAGACAGGAATTAGGTATGGAGGTAGCAATTCTTGATGCTTTGACTTCTCAGTTAATCTTTCTACCCTTTTGATCATCTCTGGCCCATATCACACTTTCTTTTTCTGAGTCTAGAGCCTCCTTGAGGTTTCACTGGGTTATCTCCTTCCTCAGCCCCAGCCGTCTCAGAGAACCTCTGGGGCTATGAGCTGCCTCTGCTTAGCTTCATCAGTCAGTACTTGGTTATTTTATGGCTTCCAGATGTTTGTTGAAATCCCTCAGGCTCCTGATAGTCTTTTAATTGTGGTGAGTGTATATACTTATTTTTAATAACTGTAATGGTATCTTTCATGTGCGTTGACCACCATTTTGAAATAGTATCTTAGTTTGGGCTGCTATAACAAAGTACCATAGATTGGCTGGCTTATAAACAACAGAAATTTATCCCTCACGGTTCTGGGGGCTGGTTTATAGAAAGCTGCTTTCTTGTCATGTCCTCTCAAAGGTGAAGGGGGTGAGAGAGCTCTCTGGAGTCTTCCTTTATAAGAGCACTAATCCCATTCATGAGTACTCTGTGATTTAACAACCTCCCCAAAGACCCACCTCCTAACACCGCCAAATGAAGATTTCAACATATAAATTGAGGCAAGAGAGACAAACATTCAGTCCGTAACAAATGAGAAGTCCTTTCATTTGTTCATTTGTCAGAAACACATCTGAAGAATTAAATCAGAAATGCAGTTAGCCAGGAAAAAAGTTTAAATAAGGGCACAATAACTGCAGTGGGTATTGGATTTTGGGCAAACTAACCCTCAAAATAACTTGGCTGGGAGAAATAAATTGGGAGGTTGGGATTGACATACACACCCGACTGTATATAAAATAGGTTAACTGATAAGGCCCTACTCTATAGCACAGGGAACTCTACTTAATACCCTGTAATGACCTATATGAGAAAAGACTCTAAAAAAGAGTGGATATATGTATATGTGTATCTATAACTGATTGATGCTCTTTGCTGTACCCCTGAAACTAACACAGCACTGTAAATCAACTACACGTGTGTGCTAAGTTTGTTCAGTCCTGTCCGACTCTTTGCGACTCTGTGGACTGTAGCCAGCCAGGCTCCTCTGTCCATGGGATTCTCCAGGCAAGAGTACAGGAGTAGGTAGCCATTCCTTGCAGCAGGGGATCTTCCTAACCCAGGGATCGAGCCCACGTCTCTTACATCTCCTGCATTGACAGGCAGATTCTTTACCGCTGGTGCCACCTGGGAAGTCCAAGTCAACTATACTTCAATAAAAATAAAAAAGAAATATCTTGGCTGGGAAGAGGCAATGAGCTGAGTTTATACAAAGACACAAAGATTTCCAGCTACAGAGGCTGTAGTTGTTCATCGTAGAGCACCCCAAGACACTCTTCAAGCTTTGTTGCTAGAAGAAAAGATGCATCTTGTGAAACATGAAAGTTAGTCATTTAAAAAAATCTGTAGAAGTAGTGGGCGCTGTTCCTTATCTTTAATACAAATATAAATGTACACGTGCTTCTTTTGTAAAATCTTGTCTGCCCTTAGACACAGGAGTTTCTGTTCCTTTGTTTCTATAACTTTTATTCCTTAAGAGAGAGATTCTCATGCTTCTCTCTTTTCTTGTAAGAAGCGTCTATATTCTCCCTTCCCATTTCCTTAGTGAAAAAAAATCTCCAAATTCCTATTGATCATTCCTTTCTCGTATTTGTCCCATAGTTCCTGAAAATGCATAATGGATACCATGAAGGTGGAAACAATAAGATGCTGATGTCCTTTATCTTTGCTCCCACAAGGGGTATCCATTGGTGCCTCAGAAGAAACTCTGAATTTATATTTCCGATCTTACCATTCCCTCATTTTAATCGGAAAAAGTAGCTGTCTCCCTGTGAATAATCCCTGGAGATCTTCTGCACATCTCCCACAGTTACCCCCAGGTGGTTGAACCTAACACAGAATCTTATCAGCCTATCCTGATGTCTAGCATAATACTAGGAGCCCCATAGTTGCTAAGCTGTGTCCAAGTCTTTTGTGACTCCATGGACTATATAGCCTGCCAGGCTCCTCTGTCCATAAGATTTCCCAGGCAAGAAGACTGGAGTGGATTGCCAGTCCCTTCTCCAGGGGATCTTCACGACCCAGGGACTGAGCCCGTCTCTCCTGCATTGGCCGGCAGATCCTTTCTGAATGAGCCACCAGGGAAGCCCTGCCCAGTAGTACTGTTGCGTATTCATTTGTGTTTGGTTTGGGGTTTTTTTTTGGTAATGGCATAGAGGTAATTGAGGATAGTAAAGATCCCCTGGTATCTGATCTCTGTATTCTGTAAGTCGTTTACCAATTTAGAGCCTTCTGGACAGTTCTAGATCAAAGAGCAGGCCCTCTGAGGCCATGCCCCAGAAGCAGATGTGGAGAGTGCTGAAGTGTCCTGAGCTCTGAGGATGCCAGCCCCCACAACATGTTTGTAGTTTATTTCTGTTATTGGCCAGAACAGATATATTATCAGCTTATTTCAGGTGCCCTTCTGTCCAAATCCGCTTGCTCCCATCTCTTCTGTAGCCTGCTGGAGGTAAGGAGTGTGTTGAGTGTGGCTTGTCAGTTGTCTACTGAGAAACAATGGGCCTAAAAAAGGACACTTGGGGAATTTAGGGCTCTGTCACACTGGTTCTGGGACCGGGCGTATCAGTTGACTGCTACTACCGGATGAGAGAAGAACAAGCAAGTTTTGTCTTTTTCTCTGGAGGACCGTGTGTGGATGGTTCCGCAGACGTGGGACAGCCATGGTGCAATGCTGCGATGTTAAGTACACTCGTGTAGCTGGACGCTGGTTAACACGTAGAACATTTCTTCACCTGCGTTTTCTTCTGCCCAGTCCAATCAGTCTCCAACCCCATTGGCAAACACTTTCTTATTTATATACCATTGCTTCTTTTCCTGTTCCTGAACTTCATCTAAATGGAATCACAGAGCAAGTACTTCTTCGTGCCTGACTTTCCCTCACACAGCAGTTTCATATTGTTGGGTGTATCCTCCATCATTCTTCCTCCTCCCATGCTGGGGAGGAATCTCTTGTATGAATAGACCCAAATCTTCGGTCCAGTTCTCCTGATGATGGACGTTTGGATTGTTTTTCCCTTTTTGGCTGTTATGGATATTAAGCTACTGTGAACCAGCTCTCTTTTTCTCAGTCTCATTCCTGTTTGAATATCAGTTTAGTCGCTGAACCAGACAGCAGATACATGTTTACCTTTATAAGAAACTTCCAGATAGCTTTCAGATGATTATTCTGTTTTATACTCCAGCCAGCAGTGGATGAGGGTCCCCGGTCCTCCAGTTCCTTCTTAACAGCTGTCTGTGTGCATGTGTGCTCAGCCCTGTCTCACTCTTTGCGACCCCATGGACTCTAGCCTACCTGGCTTCTCTGTCCAGGGAATTCTCCAGGAAAGAACACTGGAGTGGGTTGCCATTTCCTTCTCTAGGAGATCTTCCTGACCTAGGGATTGAACCTGGGTCTCTTGCATTGGCAGGCAGAT

>LNC_001782

CATACCTGAATGGTCTAGTGGTTTTCCCTACTTTCTTCAATTTAATTCTGAATTTTGCAATAAGGAGTTCATGATCTGAGTCACAGTCAGCTCCCAGGTGGAGGATGGTAACTTCAGGCTGAGCACGAGATTCCTGGAACACCGCCCTTGTTACCCTACCACCAACCAATCAGAAGAAAACCACACACCCCGCAACCCCTAACCCTTATTTTGTTTTTAAAAGCTTGTCCCCAAAAGCACTGGGGATTTTGAGGTTTCTGAGCATGAGTCACACATTCTCTTAGCTTGGCTCTTGTGATAAATCTCCCTCTGCTCCAGACTCCAACATTTCCATTCATTTGGCCTCCCTGTGTGTCAGGCACAGGGACTTGTGTCCCATAACATCTTCTTTTTCTCTTTGTGGTCATGCTCTGGTGTGGGTCTGTTTGCAATTACTGCACTAGGCATGAGAGTACTCAATGGGCCCTTTCAATCTAGCAACTCATTTCCTTCGGTTCAGAGAAAATTTCCTGAGTAACTTCCTTGATAGTTTCCCTTATTCTGTCTCTTACTGGAACTCTTATTGTCTGAAAGTTACAGCTCCAGGATTGGTTCTCTTACATTCTAAATTTTCATCTTTACCAACTTTATGTTGTAATCCTCCTATTGTGTTTTGACTTGTTTATTTTGATTTATTTTATTTCTATAATTTTTTAAATTTCCAAGAGCTATTTTTTCCCCCCTGACCATCCATGTTAATATTTAAAGAAAAACTCTATTCTGGTTTTATGGTTAAAATATCTTTTCTTACATCCGTCTGCATTTTTCCTTTTTTATTTTCTTCTCCCTGTAAAGACTATTTTTCTGTACATTGCTGTTTTCTACTTTTTATTTTAGCCCATATTTTTCATATGAGAGACTCTCTTTAGATGTACTATGATTCTTGATGGTCTGCTTGTAAGTGGGAAACTCTTACAGCGCTCATAGGACATACCACAAAGCTCTAAAGTAAAACAGTCTGGTATTGGCATATGAAGAGGCAAACAAATAGAACAGAATGATGAGTCCAGAAGTAGGTCCAAGAATAAATGGAAATTTAGTGTGTGAGAAACAGTAGATCTTGATTAATGGGGAGACAGCATTGAAACAACTGAATAGCTATTTGGGAAAAGACAAAGTTGGATTCATTCCTCATACCATACACTCCAAAGCATGAAAGATAAATGAGATGAATAAACTTCAAATGCATGCAAGATACATACAAGCGCATACAAGTACTAAAGAGAAAATATAGGTGATTCACTTTATAACTTGAGAGTAGTGAAAAAAAAAAAAATAAAATAAAATACAAATAAATAAATAAATCCAAAAGCAGTGAATGAAAAGATTAATTTGACTATACAAAAATAAAAATATTTTGCGGACAAAAGATCATATTTCTACTAATACGCAAACCACTTAGCCAAGAGTCTTAGCCACTGGACCACCAGAGAAGACTCCACATCCAATCACTGAAGGGTATGAATAAAACAAAACAGCAGAGGAAGGGCAAATTCCATCTCTCTGCTTGAGCTGGAACTTTCACCTTCTCCAGCTCTTGTCCGCTGGAGCTCCTGGCCTTTGGACTCTGACTGGGGATTAACACAATCAGCACCCCCACCCTACCTCCTTTTCTTGGGATTTTGAGTTCATCCCAGGGCTTATACCTTTGGCTCCCTTGGTTCTTGGGCCTTTGAGCTTAGACCAGCTTTCCTGGATCTCCAGCTTACAGATAGCAGATTGTGGGATTTCTCAGTATCCTTGATAATAAATCTCTTTAGATATTGGTCCTGTCTCTCTAAAGAACTCTGACTTATAAATGGGAAGTGTTAAAAGAACACAGGAGTAGCTTGAAAGGGCTCACACTAGCCAAATTTCTAAGAAGAGATTAAGCATCAAATGTGGCTATACAGTATTGTAATGACTGGGAATTGTAACAAAAATTGAAAAAGAAAATAATTCCCTGAGTCCAAAGTGACAACCAAAGATAAAGATAAAATTTAATTTACCACCATTGAGGAGAATATTTAAACATTTCCTTTGGGCTTCCCTTGTGGCTCAGCTGGTAAAGAATCCACCTGCACTGTGGGAGGCCTGGGTTCAATTCCTGGGTTGGGAAGATCCCCTGGACAAGGGAAAAGCTACCCACTCCAGTATTCTGGCCTGGAGAAGTCCATGGACTGTATAGTCCATGAGCACGAAAAGAGTCGGACACGACTGAGTGACTTTCACTTTCTTCTATAAATTGATCGAAGGAAATAGTTAAGCATTTACCCTGATGTTCAGGAAGAACTGTAGTTCACCCCTATTGATAAGGGAGAGCTTTTCTTTATAGAATGTCAGCTAACACATGTAGAATAAATGATAGAAAATCACCATTTTGCAGCTCCTCCAATGAAATAATCATTTCAAAGAAAGATCTTGAATGTATGCTAATTCATCAGGTTGCCAAGGAACAAGAATGCCAAGGGATCATATGCTAATTACAAAGGAAAATAGCATGTCTTCACAACTAAGAGACCTCCTAGTCACCATCTGGACAAGTGAGGAAACTCAGGGTCACTAACACGGGGACACCTGGACACTGCGTTTCTCCTGATGTGATATAATGTGAAGTTCCTGCCTTCTCTTTAGCACATTCTTGCCAGAAATACTTTAAACATAGCCTGAACATAACTGAGTCTTTAGTTGGATTCCAATTGATGGGAAATACAGAAGACAGGGAAACAAGGTTAATGACACTACAAAGAAACCATCAGATGGTTTAAGAATGTGGGACACTCTGCAAGACAACTGGCTTGGTCTTTTCCAATAGTCAATGTCATTTTTTGAAATGTGGGAATAATTTAGCGAAGACTAAAGAGTAGTGACAAACACGGGCTATGCATGAAGCTTGACTGAATCTTCTTTTTTAATTTTTATTTTACATTAGAGTATAGTTGATTTGCCTTGGTTCATGGACCTAACATTCCAGGTTCCTATGCAATATTGCTCTTTACAGCATCAGACCTTGCTTCTATCACCAGTCGCATCCACAATTGGGTATTGTTTTTGCTTTGGCTCCATCCTTTCATTCTTTCTGGAGTTATTTCTCCACTGATCTCCAGTAGCATATTGGGCACCTACTGACTTGGGGAGTTCCTCTTTCAGTATCCTATCATTTCTCCTTTTTATACTGTTCATGGGGTTCTCAAGGCAAGAATACCGAAGTGGTTTGCCATTCCCTTCTCCAGTGGACCACATTCTGTCAGACCTCTCCACCATGACCCACCCGTCTTGGGTGGCCCCAATGGCAT

>LNC_005777

GGTTGAGAAGTGCTTGCAAACGAGACTCTCAACCAGGGCTGAACATTCTTGCAAACGAGATGTTCTGCCCAGTGTAGGGAACTAGGTTTAAAGAAGATTGAGAAGTGCTTGCTAACGAGATGCTCAGCTAAAAATCCTGTTTGTTCCCGTGGGAAAAGAACATTGCATTGCACACAAGGATGTTTCTCTGATTCCTCAATAGGAACAGACCTTGGGACAAAACGGATTCTAAGTTGATAAGGAAGTTCCCCAAAACAAAGTCTTAGCTGCAGTAAATAAGCCAAGGTAAAGGTTATCTCGCCCTGCGCCTGCGCACTGTATAGTCTCTGAATCATAGTGCTTGGCAGCTTGCCCTG

>LNC_003630

CCCAGGTCTCCCGCATTGCGGGCAGATTCTTTACCAGCTGAGCCACCGGGGAAACCCCAATATGAAGAACAATACTCGATTAACCAATGTGTAGCTGAGGCTACTTGTGTGGGGGTAGTAGAACCAAGAATCAGTATTTGAGAGAATGTGTGTACATAATACTGGAAAAAATCAGGTGTATAAGTCAGAAGTACTGAACGCATCTTAATATTATTCTCTTTCTACTCTTTCTTTTTCTTCCTTTAGCGTTTTCTTCTTTCTTGGCTTTCCTTCACTGGAATGCTCCCACATGCAGTTTTCCCATTTTTCTTTTTTTTTCCTCTGTTTCTGCAATCCTCGCCTTCATACTCCATGCCTGTTTCTGTCACTACTCCGCTTTTTCCCACTTTGACCTAGTGGGAAAGTAAGAAAAAGTTGCATAGGGATTGTCAGAGTTGATGGCAGATTGACTCATGTTCCTTGGTTGTAAGTTGAAAAATTATGGAGGACTTTAAAAGAATTCGCTTATTTAAAAAGTGTAACTTCAGAGGCAAAACGAATTTATGGCTGAGCGAGACAGAGTATGGCTTCATTCAGTTCAGTTCAGTTCGGTTGCTCAGTCGTGTCCGACTCTTTGTGACCCCACGAATCACAGCCCGCCAGGCCTGCCTGTCCACCGTATGGCTTAGTGGGTGCCTGTTGACGTGGATTCTGAAATTACTCCATCTGTGTCACCTTGCACAATTCCCTTTCGCCTCTTTGTATCCCAGTTTCCCTGTGTTCCCTTTGTATTAAAAGGATGATGATAATAGAAACTGTTTCTCTTTTAGGGTTGTTCTGAAGATTGAATGAATTAAAATTAAATACTATGTTTAATACTTTACAGAAATTGAATTTCCTAAGGAAATAAAAACTGCATGTGATACAATCCTTGTTATACTGATTACAGGGTTAATCTCCATAGACTCTGGAGAATGTAAATTCTGTGAGGGCAAGAATTGTTCTTCGGGGATTTTCCTCCTCTGACAAAGTGTTTTTGTCTTTGTAGGATGGCAAAGGAAATACATCACCCATTCCTCAGCTTAAAGTACTATCTGTGATTGTGCATGGGAATTCGTAGGTGAGAATTGTGGAGTGGGTTGCCATGTCCATCTCCAGCGCCTCTTCCCGACCCAGGGATTGAACCCCGGTCTCCTGGATTGCAGGCAGATTTTTTCTCATCTGAGGCACCAGGGAAACCTGTATGTGATTGTATTCTGGTATAATTGTAGAATCAGAGTGTAGTCAAGTGCTTGGTCCTATTTGGGGTATCTGGTTAAAATGAGCCCTGGTTAGTAGACAGCTATCTATAATGTGAATTTCTATAATGGAGATTAATGTCTGCCTTGTAATTAGTAAACTTAAAACTGGAAAATAGCCTTTAGAACTGGTTACCCAACACAGAATCCCTCCACCAATTTTCTTCAGCTATCACACAAGTCTTCAAAGCAGTATCTTCCCCCCACCCCACCCCTGCCCCGGGGAGTCGTTATATTTGAGATAGAAACTCATAAATGTGTTGAAGGGCAATTGGTATTTCTTTGTTTTTATTGGGCAATTAAAGAAGGTAAGAAAAGTTTAGCTGTCTTAATTGTGGTCAGGTCTCACTCACCTTAGTGTTTTCACTAGAAATGGACAATTAAATGATACCTGAGAAAACTGCTTATATCTTGTTCATTATTAAGAATTCGATATATTTAGAAAAAGCTCTTAGTATTGACTATAACATAAATGTGAACAGAGTTTTGTCTCGATTGAATTTTCAGACCAAAATTTACTTAAAATGAGATAGTGGAACTGCACTAAAATTAAAAAGTATTTGGCCTCAGTCTTTAAACCACTTACTTTATTGAAATGATAATTTGACAAAGGAGGATGACAGCGTGTAAAGTGCCCCCCTAGTACTGGAATTAGTTTGAAAAGTGCCTAAAGGTGTTCAGGGAAATTTGTTTGCTTCCCACTTTTCTCCTGTTCTTTTTCTGTTTTTCCATTTAAAGCATTTTTAAGCCTTTTAAATTCAGTGCCTTTAATTACAATCACAGCATTGTATAACCATCACCACTATTTCTAAAATCTTTTTCATTGCCCCAGATAGAAGGCCATTAAATGATAACTTCCCTTTCCTTCTTCCCTGGAGTTCCTGGAAATCTCTTAATTTACTTTCAGTCTCTGAATCTGCTTATTTTAGATAGTTCATGTAGGTAGAATCCTGCAGCACTTGTCCTTTTGTGTCTGGCTGTTTCACTTGGAGTAGTATTCTCAGGGTTCATCTGGGTCGTATGTACCAGAATGTCATTCCTTTTTGTAAAATGGCTGGATGATACTGCATTGCATGATACACCGAATTTTGTTTTCTGCTCCTCTGTTGATGGACATTTAGATTGTTTCTACCTTTTGGACGTTACAGTGTTGCAACATGAACATTGCTGTACAAGTATTTTGAGTCTTCACTGTTTTCAGGCTTTTTGTGTGTATGCTTAGTGGAATTGCTGGATCGTTGCTTTTTCTTTTTATCGATTTCCTTGGGGCAGAATTTAGGTGGATCTGAACAGATCTCTAAATAGCAGAGAAAACCGCTGAGAAATGAGTTTGTCCCTTTGTGAAGAATTTGCGTTGGCCATTTCCACCATGAAAACCTCTTTCTTTCTGATAGTTCCTTATAGTGATTTTTTTTAAAAATTGTGGTAAAATATACATACCACAAAGCAGTGTTAAGGGCAGTGTGTACAGTCACAGGGTTGAGCAGCCTTCATAAAGTCCAGCTCCAGGACTTTTTCATCTTCCTAAACTGAAGTTGCCCATTAACCCATTCCCTCTTTCTTGCAGCCCCTGCACTCACCGTTTTACTTTCTGTCTCTAGGGATTTGACTGTTTGATTTGTTTTATTTGGCTTTGGTGCAGAGATCTTAGTTTGCTGTGTGGAATCTTAGTTTCCCCACTAGGAATCAAACCCAGGCCCACCACTGCAGTGGAAGCGCAGAGTCTGGGCAAGTCCCCATATGTGTGTATTTTAGAAATAAGTGATTTAATTAAATTTACAATTAAGTGCTTATTCAGTTTATGGAATGCTTATGTTTAGCATTCTGTCAGGTACTTGACAGATTCTGATAGGAACCTTGTGAGTTAAGTTGACATATTTATTGCCCTTTCCAGACTAGGAGATTTGGGATTAAAGAATCCCACAGCCAGCCATATCTAATATTGGAGCTAGGCCTTTAATTCAGAATTTTGGCCTGATTACAAAGCTGCCGTTCATAATTGCTTGGCTCTGTTGCCTGCTTTCTGTGGACAATACACTAAAAAGAAAAGATGGCGCTTTTAGGCCAGTTACTTGATTTTTATTTCCTCCTCCAATTCATACAAAGTTAGAAAAAGACAAGTGAGCCAGTTTAACAAGTAAACCAAATGTTTTTTAAACTTGAAGTAAAGTAAAAGGAATATATTCTTTGTGTATTGGTAGAGAAAGCTGCTACTTTCAAAAAACTCTGGACTATGAGTATAAAAATATACTTTATACATGTGTGAAAAATTTCAAACTAGTGCTGAGGAAAGTTGTGGATTTTCCTGAATAGTTGATAAGGTCTGTGTTTGTTTTACAAAGACTGTTCTGGATACTGTGAAACTCAGTATTGAACTTGTGATCCACTATTGGGAAAGATCAACTTTCTCTGGCTTCATTTAATTATTTTATCATTCTTTCCTTCACCTGTTGTGTTTTTTTTTTTTTTCCCCCTAGTTTTTATTATTTTACTTATTTGACTGCATCGTGAGGCCTGTGGAATCTGTTCCCTGAGCAGGGATCAAACAGACCTCTTGCTTGGAGTCTTTAACAACTGGACCACCAGGGAAGTCCCCTGGCTTTTTTATTTCTAAATATTTTATGTATTCATTTGGCTGCAAAGGGTCTTAGTTGCCCCGAGGTATAAGGGATCTTAGTTTTCTGACCAGGGCTCGAACCCCTGTCCCCTGCATAGAAAGGCAGGTTCTTAACCACTGGATCATCCGAGAAGTCCCCCCTGGCTTTTTAAAAAAGGGAAAAATTTGACTGTTTTTGTTAGCTATCTCAACAGTTTTTTGGAAATAGGGTGGAGAGGGTATAATGTTTTAAGTATGAAAGTAAACACTTGGAGATCATTTCAAATCCTAACCTTTGCAATATATTTTATTTTGTCTTGAGAGATTGCTTGATGTTTACTGATAAAATTACAAACTTTATTTCTGCAGAAATAAGAATTTACGCTAGTGCTAAAGAACCTGCCTGCCGATGAAGGAGACGAGATTTGAGTTCCATCCGTGGGTCGGGAAGATCCCCTGGAGGAGGAAGTGGCTACCCACTCCAGTATTCTTACCTGGAAAATCCCATGGATAGAGGAGCCTGGCAGGCTACAGTCCATAGGGTCACAGAGTCGGACGCAACCAAAGCAACTTAACACATCTTAAAATGTAAACGTAGCCTACTATAGAAACTTAAAAGCATTGACTTAAAAATGAGCCTTGATCCATCTTGAATTAACCAGTGTGTCATGAGTTCTGACTGTGCTGAACTGGATGGAGGACCTGGCCCCAAACCCAAACACAGTACTGTTGAAACTAAAAGACCCTGGTACACCATTTGATTGATACATAGTCTGTACGAGAAGTTTTTTCTGACAATCTTTAGATAAAGTTGTGTGCATGTTTATACCTTTGACATGAAATTCTAACTACTCCTTCCTTCAAATGCAAGAAGCAAAAAAAAAAAAATCCAATTTAATTTAGCTAATCGGGACAAATTCTAATCAGTAATCACAGTGTAACATCCTTCACTCTCCCAGTTCTAATACTCCACTTGTGACTGCGAAAGCAAAAAAGTTAGAACCCCTTTGAAGTCAGGTAAAAGTAGGAGGAATTTTAATTAGTGGCTATGTTTTAATTAATATTTTAAAAATTATTTTAATATTTAATTACTTTAAACAAAATAATTATAAGAAGAGCAAACAAACAAAAATTTCCCACATACCCTTCATCCTGCAAGCCCGTTCATGTTTTGCCAAACACCCCTGTATGTAGCAAAAGGCCTAGTCCAGCAGCGCACTGAGTTCTCCGTCCAGGGATTGAACTGTGGCCTCGGCAATAAAAGCTCAGCGTCCTAACCTCTGAACCACTAGGGAATCCCCAATATCACACTTTTCATCCAGTTGTCCCATCTCTATAATATACTTCACTGTAGAGCGGTTCCTCTGTCTTTGATGTTCATGCCCTTGACGGTTTTGAAGGGCATTTCATAACATGACTGTCAGCTCTGGTTTGTCCAGTGCGTTTTGGACAGGAGCATCACAGTGTTCTTGGGTTTTGCTGGGTGGGCGCGATGTATATTTGTCCTGTGACTGGAGTTACTTTGAACACTTGGTTAAGATGGTAGATGATGTATGCTATATTTTGCTTTGCCTAGTAAGTGTTTTGTGAGGCAGTACTTAGGGATAATGAAAATATCTCTTGTCTTTTTAGCATTGCGGAATGCTGGGAATTTTAAAAATTTAGGCTGAGAATTCTAAACTTTTTTAGGGGTGACATTTAGTGCTTGAACCTAAAAACCTCATGTAGAGGCCTTAACCTAAGTCCTTGAAAACAATGAAATTTAAATATTGTGGGTAGTGGATTTTGAAAAATGTTAAAAATTGGGTTATAGTGTACAGTGTATAGTGTAGTTTAAAAGAACTTGGGAATTAAGAACAGTCAGATATGGATGAGACTAATAATATAATAATTCTGTGATTGTGGGCAATTTACTTTCTAAGTTATCAACTAGTCTTATGTTAGAAAAGGTCTGCTTTGTAAAATATGTCTGGCAGCTGTCACAATAAACATGTCAAGATACTATGTTAGCTTCTTGGGATCTTTCAGAAGTTAAACTAATGGAGTCAATGAATTACTATAAATACATTTTCTTTAGAAACTGCTCAGTGGACATAATTAGAACACATGGTTGGTGCATGCTCAGTCGTGTCTGACTCTTTGCGACCCCATGGACTGTAGCCCGCCAAGCTCCTCTGTCCATGGGGTTTCTCAGTCAAGAATACTGGAGAGGGTTGCCATTTTCTCCTCCAGGGGATCTTCAGGGCCTAGGGATCGAACCTGCGTCTCCTGCATTGCCAAGTTGGTTCTTTACTGCTGAGCCAGCTGGGAAGACCAGAACAAATGGTACTATGCTTAAAAAAATTTAAGATATTTATAAAATCTTTGTTCACAACCCACAGTGATTTCACTAAATGTATGAGTTTAGTTTTAGGGATGAGTAAGTTTTCTCTCCTAAAAAAAACTTTAGCTTTTAGGATATTGTAGCTAGAACACAGAGCTATTAAATACAAGTTTCAGAGGTGGAAATTTTTAGGCAGATTAATAGATTGCCAGTATGTAATATTACATGATAACCTATACTAATTTTAGTGGAACTGAATCTTGTTTAGGATAAAATCAGCTGATTCAGCTATTAGGTCCTTGCCTTTTATTTTAGGTACCATCTAAAGAAAATATATAGTATCTTAGATGATGAATTTTAGCTTTTTCTTAGTTCTCTTGATTAAAAAAAATTACTTTCATAAAATCAGAATTTTATAAAAGTTTGAAATTCCATTTTACTGTTCTTAGAATTAAGTGATACTGAAAGTTATTTTACATTTCTCGCTGAAGATGAGTGTAGTTAAGACTGTAATGATGCCTTTAGTCACAATTTTTTTGAAATCTGGCTTTCATGGCTGTTAGTAAAATTAAGTGAATTGGTAAATTTGAGACTTAGCTAACCTTATAAACAAAAAATAACCTGAGACCAGATCTCAAATAATGGAGACAACTAACAAAGATGTGTGTGTGTGTGTGGTTAGTCACTCAGCTGTGTCTGACACTTTGTGACCCTGTGGATTATAGCCCGCCAGGCTCCTTTGTCCATGGAATTCTCCAGGCAAGAATACTGGAGTAGGGTGCCGTGCCTCCTCTAGGGGATCTTCCCGACCCAGGGACCGAACGCACTGCAGGCAGATTCTGAGCCACCAGGGAAGCCCAAGAATACTGGAGTGGGTAGCTTATCCCTTCTCCGGGGGAATCTTCCTGACCCAAGAAACAAACTGGAGTGTCTGCATTGCAGGCAGATCCTTCACCTGCTGAGGCTACCAAGCAACAACCAAAAAACCAAGGAAGAGATAGCTCATCTTCAATGCTGGTACTGTGGAAGCAACCACAGCACAGGAAAGCCACGCTGCATTTTGTTGGAGTGCGGGTCGAAAAATTAAGTTTCGGTGGAATACTTTCCTGGTTAATTAAAGTATTCCCCCCCCCCCTTTTTTTAAACTCTTAAGCCTGTTTTATTGCAGAATTGAATACCGTATTATCTGTCCTAAATATTGTCTGTAGCCTTCCATGGCCAATATCCTAGGGGTAAGTATGTTAATTGATTTTTAATGTTTTCTCAGTTTTAATTGGTCCAGGTATCTGCGGTTTAGTAATTATGGCTCTAGAAATAAAACAGTGAGTTGGGAAACTTACTGGTTTTTGTAGTCGTAAAGTTTATTTCACCGTGCTTTGCTAAATGTACAGTGTGGCTCTTAGTTGCCTATAATAAGTAAAAATAACAGAACTTAAACTGACTATAGAAAACTGAAAATGAAATTTTTGAGAAGAAAACGTTGAGCCTGGTGCCTGGCTGAATGCAAACCCATTTCTTTCTAGCTGACCATGGGTCTTGGTTTTGGGATTTTACTAAAATAAAGTGTTGCTTAAACGACTCATGGCTGTTTGCATTGTTACTGGTGATGTGTGTGCCTGACCGTGCCTGTCAGCAGAGTTTCGTGACGTAAAGTTACGCCCTCGGCTAGACAGTCGGTCTGCAAATAGTCCATATTTATTTCTTTGAACAACATTCTTTGTTTTTTTCAGAAAGTGCTGTTGTATGTTCATATGTACCCTTCAGTGTCCC

>LNC_004460

TTAATTTCATGGCTGCAGTCACCATCTACAGTGATTTTGGAGCCCCAAAATTAAAGTCTCTCACTGTTTCCACTGTTTTCCCATCTATTTGCCATGAAGTGATGGGACCAGATGCCATGATCTTCGTTTTCTGAATGTTGAGCTTTAAGCCAACTTTTTCACTCTCCTCTTACACTGTCATCAAAAAACTATTTAGTTCTTCTTCACTTTCTTCCTCAGTTGATTCCAGCTTGTGCCTCCTCCAGCTCAGCATTTTTCATGAAGTGCTCTGCATATAAGTTAAATAAGCAGGGTGACAATATATAGTCTTGACGTACTCCTTTCCCAATTTGGAACCAGTCTGTTGTTCCATGTCCAGTTCTAACTGTTGCTTCCGGACTTGCATACAGATTTCTCAAGAGGCAGGTCAGGTGGTCTGGTATTCCCATCTCTTGAAGAATTTTCCACAGTTTGTTGTGATCCAGAAAGTCAAAGTCTTTGGCATAGTCAATAAAGCAGAAATAGATGTTTTTCTGGAACTCTCTTGCTTTTTCCATGATCCAACGGATGTTGGCAACACCTAAGCAAAGATACTGTCATCTGTACCAGCTAATCAGCTAAAACAATGTGTCACATTAAAGACCCAGATTTTCTCAAATCCATAATGTCAAACATGCCATCAGGACATGGAAATTAAGATTTTAGATATATGATTTCTGTATATATATGCCTAATTTTCTCTGTCAGAAGTGGCTTACAGTCACTCTCTTCCTTCAGAAATATGATTAACAAGGTCGAACTATGTATGGTAATTTAAAATTGTCAAAGTCAGTGCTGCCTGATAGAACCTTCTGCCATGAAGCACTGTTCTGCATCCGTGCTGTCCAATTTGGTAGCCATGAACCACACAGGGCCAGTGGACTGAAGAACTGAATCAGTAATTTAATTTTTATTTTATATACTCAGTGGCAGGTGTTTAGTAGCTACTATACTGAACAATACATATACAGGATACTGAAATTATTTAATATTTTCATTTAAAAATACTTTTAAAATGGAAAAAAAGTGGTTGCTTTAACAAAAGCCTTAAATATAGATTAGCAGAGCAGAGCTGAGTGGGCATTACTGTTGTCCTTATATGAACCTTTTCTCTATCATCATAAAGTAAAAGTAGCTCAGTCGTGTCTGACTCTTTGCAACCCCATGGACTTTACAGTCCATGGAATTCTCCAGGCCAAAATACTAGAACAAATAGCTTTTCCCTTCTCCAGGAAGAATCATCATAACAAGTTAATACAGTCCTGTTTTGCACAATGTACCATCTCATAAATCTATACAACCTAAGGAGAACATTGGTATAGACTGCCCCCAAAACACAACTGAGCAATCATTAATTATATAAGAATTAAAGAAGTGAATGAAATAAATGTGGTGTGCAAAGAGGAAGAATGCAATGGGAAGTCTGTGGTGAGAATGAAACTTCAAGTGCTGAAAAAGTAAGAATCAAAACTTTTCGGAAAAAAAGATATTCCAGGCATAATGTGAGGCCTGAAAAGATAAACCAAGGTGGAAGTATGTGTAGTTGAAAAACTGTATTTTCCTGAGAAGACAGTGGATTTATCTGAGAAATATAAACAGGGGAAATAAAATAGGTGAGGACTTGAAAAACAAATGAGTCTTTATTCTATTCTTATTTCTACATCTTCAGGTGACTCTCTTAATATATATTTGAAAATTAAAAGGACTTTTAAAGCTGGTTGTTTTGAAGTCTCTCAGAGCTGTAGAGATGTATTCAGACAAATTTTTTTCCATGAGTGCTACTCTGATCCAGGTTCTACCCTAAAAGGAAGAAGAAAGAATCAAACATAAAGAAAAAATTAGAAATTAAAAATTTTGTGTTCACTCATCAAAAATATAAGCATTTTAACATGCAAAGAATAACTCACTCAAAATCTGTATTTGATAAAGACTAAAAAATATTTAAGTAGATAAGATGAGGTAAAATTTAATTATATATAGTATATATTTTGTTTCTCTAAATGTGTAGGGTAGGGCATGTCCAATTAACTTTCTAGCTTCTGAAGGAGCAGAACCATTAAAATTGAGTTTACTT

>LNC_004835

TCCTTCACTATCTTCCAGAGTTTGCTTAAACTCATGTTCACTGAGTCAGTGATGCCATCAACCATCTCATCCTGTGTTGCCCCCTTCTTCTCCTGCCCTCAATTAATAGTTTGATTTAAAAATTATCTTTAGACAATATTAAAGTTCATGAAAGATCAATAAATTCTTTTTGCTAAATTGTGTCTTTTTAGCCCTATGGGGGAAAAATATAGTAAATTCTTTTTCAGCAACTGCCTTTCAAAATCTTCAGAATTCTATCATAACTATCAGGCATCCACATGGAGAGACCTTAGCTTCCCAGAATCAGACTTGGCAGTCCTAACGATTGGCTTGGATACCGTGAAAGAAACATTCCTTACAGAGATCACACAGGGAGAAATGCTCCATCTAACTCCCTATATTTAATCTTCACAAAGATTCAAATTATCTTGAATCCAAATAAGTCCTCTTGGAAACAAGACACAACAGATGAACCATGAGCAAATTAAACCTACCTCCAAGATAATAATTATCTCCCATTTAATTTGCCTTCCTTTTAGGCAGGATCTGCCCAGACTAAATTAGAAGTTCTCTCTGAAAAGCTGAAAAAATTGATTTATCAAAAAAATTAGAAAGTGAATTTGATATTTAAAAGCACCAGTAGACTAGAATGCAGAAGGTAACCATCCCACGCAAGTCAGGCCTTCTCTGACCATCCTGCCTACTTACAGGTGATAATTTGGGCCATGCTCAGAGGGGAATCTGAAAGCATGCAGAGACACTAATATCTACACCACTTGTGGATGAACTAGGGTTATTAGTCTGCAGAAGCAAAGCCCAGAGGCCAGGTGGGGAGAGTGGGGCATGGGGGTGAGTTCAGATATTTAAAGGTTTGTCACATTGTAGAGTAAGTCTGTGCAGCCTGATGGGGTGAAATGGAACAATTGTGTGGGAAGAGATGACAACCGAATCGTTATAAAGGGGAACTCTGAACAACTAGAGATGCCCAAGCATGAACTGGGCTGTTTCTAATGGTAATGAGTTCCTGTCTTTGATAGTTTCATTCAATATTCTACATTCCTTTTATCAAATAATTACTCTGAAGGCTCAACATAGGCTACACACAGGTAAATGAAATAACCATGGTTCCTACTCTCATCCAACAGAGGAGACTGGGAAATTTTTCCTAACCTCAAGAAACTGTGAAGTAAAATCCATATTTTTTTTTCCTACATTTGAAGAGCCCCAGATATGTTTTTATTCTTACCAAGACTGAGCCAACCAGATTGATAACCCTTAGTTATGAAACAGCTGAAGGCTAGCATAAAGAATATTTGGGATACTATTGGCATCAAGCCATTGAAGTTATAACTGTTGGTCTTCAAATTGCATTAATAAAGGAACCTCAAGTCTGTCTTCCTAAAGTGAATTATTTAGGATCATCCTGTTGCTCTTGCAACAGGTTTCATGGTTAGAAGATATTGCGCTGGCCAAAAAGTTTGTTCAGATTTTCTGAATAACCTTTTAGCCAACCCAAAAGTTTTCCTTTGGAAAAATGGAATAACAGTATTTCATAATTAGAAAGAAACACAAGGCTGGTATATACTGAACAAGGTTTTGTTTTTAATGGTTAACATTAAAAAACATTAGTCTCAGAATTGAATCCCTTAATGAACAAAGCTGGTTAAGTATTTACAATCTATCCTTGCCTATTAATGTTTATTATTGAAGTTCATTTCTAATTATTTACCTAATCAGCACATTCCAGGACATGATTAGTTTACCTTAATCTGAATTTAACAACTATGATCTTAGACTTGGATCACATGATCACTTGAGAAAATGACCTGTTGTAGCTAAAAGGCACCTTCGAGTTTGCATAGATTGTCAGGAGGCAGAGGAGAGAAACTCTAGGGAAATTCCAGGGGGTTAGACTCACTGAACCTCCCGGCATGGCTCCAGGCAACAGAGAACTCACAAATATGCCTTCATGGAAAATATTCTGACACTAACAAAAGCTCAGCTCACTTTATTTTTATTGCTAAGACTAATCCCCAAAACTTCTAAAAGCAAGCTATGTATTAATCAAAGCTAGAATGCAGAAGAGAAGAAGAAAAACAAAACAACGTCAGGGACTGAAAAGTCAAAAGCAGCAGGAGAGAGCCTGCTACTCAGTTTCTAGAATGAGAAGGAGTGTTTTATGGAAACTTCTGGATTTAGATCTACTTTATTTCTACTTAACAGGAGAACAGGCTTCCCTGGTGGCTTGGTGGTAAAGAATCTGTCCGCCAACACAGGAGATGCAAGAGACACAGGCTCAATGCCTGGGTCAGGAAGATCCCCTGGAGAAGGAAATGGCAACCCACTCCAGTATTCTTGCCTAGAGAATCCTCATGGACAGAGGAGCCTGGCGGGCTACAATCCATGGGGTCTCAAAGAGTTGGACATGACTCAGCACGCATGTACACATCACAGAACTAAATTGGAGAGATTTAAGGCTATGAACAATGTCTATTTATCTCACTTCATGGGAATATTCACACATTTAGCGAAAGAAATATTATTGTGTTGAATTACAAGCTGGTGATATTATGTAATGTGAAACATATGTCATATTACCTTCCTAAAATCCAAAAATCTCTCTGAATTCCAAAACATACCTGTCCCCAGGAATTTTGGATATAGGTTTGTTAGATCGGAAATAGAGTCACAACTAGTTCCAGAGGTTTTTTCAATATTTTATACAGGACTGTGGCTAATAGGGAGAGGCCATGTATAGAAAATTAGAAGTTCTCATAACTGAGTGGAAAGAGCTCGTAATTTAATGGAAAGCGAGTACCTCTATAGAGCCAGGAATTAGGGTAGAGTGTCTCGCAAAGCCCTGGAGAAGACAGTGTTTGCCCCTTAACAGCCAGTTGCTTGTCTTTCTGGACGCCTGTGTGCATGCTCAGTCGCTTCAGTCATGTCTGACTCTTTGCAACCCTATGAACTGTAGCCCACCAGGCTCCTCTGTCCACAGGGTTTTCTAGGCAAGGATACTGGAGTGGGTTGCCCAGCCCTCCTCCAAGGGATCTTCCTGGCCCAGGGACTGAATCTGCATCTCCTGCAGTTCCTGTGTTGCCGGCAGTCACCTCTGAGCCACCAGGGAAGCCCTATCTCTCTAGATTCCTTTGACAAACATTCACCCAGGCAGGAAACTACATGGAGAGAAACCTGTTCTTGAATTCAGTGCAGAATTCCCTGCGGGATAAAATATCTCATCACTATACAAGTTTTAAAATAATGATTTGATTCCTGAGCTAACCCCCCAAAAGTTTGCTCCTAGCATAGCAAAAGCAGTAGAATTTGTAATATGGGATAAGCTCACTTTTAAGAATGAATCTATGGATCTGAGTTCTTTTCACATTACTGTTTATATGAAAAAAATGTATTCTGCATCCCAAATAATTAATGTATTATATTAAAATAATTAATGTATTATATTAAAATAATGTATTATATTAAAATAATATATTTTATATAATATATAATGTTATATGTTATTAATATATTATATAATATATTATATAACACAATAATATATTAATGTATTATAATAAAAATGTATTCTGCATCCCAAATAATATGCAAAATGTCATTTAGAACACAATGCATTTCTATATTGGATGTTGCCTGTATAATATGCAGTGTTTTTCTAATTGTCATCATTCATTGTTCAAGAAACATTTACTAGGCACTATTCATAGGTAATGGAAATAGAGTTCAACAAGAGACAATGAACCAGCCCTCACACAGTTTAGATGCTGGGGGAGAGACAATAGGAAACAATGTGAAAGTAAGCTCATCTTTTTTTTTCTTTTTTTGATTGAAGTAAAGTTGATTTACCATGCTGTGCCAATCTCTGCTATACAGCAAAGTGACTCAGTTATACACACACATACATTCTTTTTTTTATATATTCTTTTCCATTATGGTTATCCCAGGAGATGAATGCAGTTCCCTGTGCTGTATAGTATTCTATTGGACCTTGCTGTTTATCCATTCTTAATGTAATAATTTGCATCTACCAACCCCAAACTCCAAGTCCATCCCTCTGGCTCTCCCCTCTCTTTCTTGGCAATCACAGGTCTGGTCTCTATGTCAATGAGTCTGTTTCTATTTTATAGAAAGGTTTCTCTGTGCCATACTTTAGATTCCACATGTAAGTGATATTATATAGTATTTGTCTTTCTCTTTCTGATTTACTTCATTTAGTATGATAATCTCTAGTTGCACCCATGTTGCTGCAAATGGCATTATTTTGTTCTTTTTATGGCTGGGGGGCAGGAGGAGAAGGGGAGGACAGAGGATGAGATGGCTGGATGGCATCACTGACTCGATGGACATGAGTCTGAGTGAACTCTGGGAGTTGGTGATGGACAGGGAGGCCTGGTGTGC
